# Supplementary material for: Estimating behavioural relaxation induced by COVID-19 vaccines in the first months of their rollout
Source: PLoS Comput Biol. 2025 Jul 7;21(7):e1013266. doi: 10.1371/journal.pcbi.1013266 (PMC12258572; doi:10.1371/journal.pcbi.1013266)
Supplement: S1 Text — In this supplementary file (PDF), we present additional analyses and results of our work. (PDF) [file pcbi.1013266.s001.pdf]

Supporting Information for: Estimating behavioural relaxation induced by COVID-19 vaccines in the first months of their rollout

Yuhan Li[1], Nicolò Gozzi[2], Nicola Perra[1,3*],

1 School of Mathematical Sciences, Queen Mary University, London, UK
2 ISI Foundation, Turin, Italy
3 The Alan Turing Institute, London, UK

* n.perra@qmul.ac.uk

Contents

1 Epidemic models **1**
1.1 Formulation for epidemic models . . . 1
1.2 R_0 calculation . . . 3
1.3 Sensitivity to the modelling framework . . . 5

2 Demographics of regions considered **9**
2.1 Contact matrices . . . 9
2.2 Spectral radius of contact matrices as a function of time . . . 9
2.3 Contact intensity and population distribution . . . 9
2.4 Validation of contacts reduction estimation: comparison with independent data from Lombardy 11

3 Impact of vaccines and NPIs **13**
3.1 Timing of vaccination impact . . . 13
3.2 Influence of NPIs on peak deaths . . . 14
3.3 Relative difference of infection in counterfactual scenarios without vaccination or NPIs . . . 16

4 Impact of behavioural relaxation **18**
4.1 Behavioural transition rate . . . 18
4.2 Fraction of non-compliant individuals as function of time . . . 18
4.3 Relative difference of infection in counterfactual scenarios without behavioural relaxation . . 19
4.4 Absolute death differences across counterfactual scenarios . . . 20

5 Model calibration **22**
5.1 AIC/BIC scores of models . . . 22
5.2 Sensitivity analysis of AIC weight . . . 22
5.3 Sensitivity analysis of BIC . . . 22
5.4 Posterior distributions of parameters . . . 24

6 Bibliography **30**

1 Epidemic models

1.1 Formulation for epidemic models

All the epidemic models studied here are based and built on a baseline which is a stochastic age-stratified epidemic compartmental model that integrates vaccination, NPIs, and the emergence/spread of a second

variant. We consider a Susceptible-Latent-Infected-Recovered (SLIR) compartmentalization with the addition of deaths. Individuals are grouped into 16 age brackets with a five-year interval (except for the last 75+ group). We use age-stratified Infection Fatality rates (IFR) from Ref. [1] and age-stratified contact matrices from Ref. [2]. The natural history of the disease is modelled as follow. Susceptible individuals (S) transition to the latent stage (L compartment) where they are infected but not yet infectious. We assume a force of infection (i.e., the rate at which S get infected) function of age, transmissibility of each strain, contact matrices, and NPIs (see below for details). Individuals stay in L for an average of ϵ^{-1} $days^{-1}$. After, they become infectious thus transitioning to the I compartment. After the infectious period μ^{-1}, individuals either recover with the probability $(1 - IFR_k)$ (transitioning to R) or die from the disease with probability IFR_k (transitioning to D), where k denotes the age-group. We also consider a delay of Δ days in reporting deaths. Therefore, individuals are moved to compartment D^o from D after Δ days, capturing the delay in deaths reporting.

We simulate the disease progression by using stochastic chain binomial processes in all models. For age group k the baseline model is defined by the following set of stochastic equations:

$$\begin{aligned}
S_k(t+\delta t) &= S_k(t) - Mult_1(S_k(t), \lambda_k, \lambda'_k) - Mult_2(S_k(t), \lambda_k, \lambda'_k) && (1)\\
L_k(t+\delta t) &= L_k(t) + Mult_1(S_k(t), \lambda_k, \lambda'_k) - Bin(L_k(t), \epsilon) && (2)\\
I_k(t+\delta t) &= I_k(t) + Bin(L_k(t), \epsilon) - Bin(I_k(t), \mu) && (3)\\
R_k(t+\delta t) &= R_k(t) + Bin(I_k(t), \mu)(1 - IFR_k) && (4)\\
D_k(t+\delta t) &= D_k(t) + Bin(I_k(t), \mu) IFR_k && (5)\\
D^o_k(t+\delta t) &= D_k(t+\delta t - \Delta) && (6)\\
L'_k(t+\delta t) &= L'_k(t) + Mult_2(S_k(t), \lambda_k, \lambda'_k) - Bin(L'_k(t), \epsilon) && (7)\\
I'_k(t+\delta t) &= I'_k(t) + Bin(L'_k(t), \epsilon) - Bin(I'_k(t), \mu) && (8)\\
R'_k(t+\delta t) &= R'_k(t) + Bin(I'_k(t), \mu)(1 - IFR_k) && (9)\\
D'_k(t+\delta t) &= D'_k(t) + Bin(I'_k(t), \mu) IFR_k && (10)\\
D^{o\prime}_k(t+\delta t) &= D'_k(t+\delta t - \Delta) && (11)\\
S^V_k(t+\delta t) &= S^V_k(t) - Mult_1(S^V_k(t), (1 - VE_S)\lambda_k, (1 - VE'_S)\lambda'_k) \\
&\quad - Mult_2(S^V_k(t), (1 - VE_S)\lambda_k, (1 - VE'_S)\lambda'_k) && (12)\\
L^V_k(t+\delta t) &= L^V_k(t) + Mult_1(S^V_k(t), (1 - VE_S)\lambda_k, (1 - VE'_S)\lambda'_k) - Bin(L^V_k(t), \epsilon) && (13)\\
I^V_k(t+\delta t) &= I^V_k(t) + Bin(L^V_k(t), \epsilon) - Bin(I^V_k(t), \mu) && (14)\\
R^V_k(t+\delta t) &= R^V_k(t) + Bin(I^V_k(t), \mu)(1 - (1 - VE_M) IFR_k) && (15)\\
D^V_k(t+\delta t) &= D^V_k(t) + Bin(I^V_k(t), \mu)(1 - VE_M) IFR_k && (16)\\
D^{oV}_k(t+\delta t) &= D^V_k(t+\delta t - \Delta) && (17)\\
L^{V'}_k(t+\delta t) &= L^{V'}_k(t) + Mult_2(S^V_k(t), (1 - VE_S)\lambda_k, (1 - VE'_S)\lambda'_k) - Bin(L^{V'}_k(t), \epsilon) && (18)\\
I^{V'}_k(t+\delta t) &= I^{V'}_k(t) + Bin(L^{V'}_k(t), \epsilon) - Bin(I^{V'}_k(t), \mu) && (19)\\
R^{V'}_k(t+\delta t) &= R^{V'}_k(t) + Bin(I^{V'}_k(t), \mu)(1 - (1 - VE'_M) IFR_k) && (20)\\
D^{V'}_k(t+\delta t) &= D^{V'}_k(t) + Bin(I^{V'}_k(t), \mu)(1 - VE'_M) IFR_k && (21)\\
D^{oV'}_k(t+\delta t) &= D^{V'}_k(t+\delta t - \Delta) && (22)
\end{aligned}$$

where the force of infection of the original strain is

$$\lambda_k = \beta \sum_{j=1}^{K} \frac{\mathbf{C}'_{kj}(I_j + I^V_j)}{N_j} \tag{23}$$

while is the force of infection of the second strain (if any) is

$$\lambda_k^{'} = \sigma\beta \sum_{j=1}^{K} \frac{\mathbf{C'}_{kj}(I_j^{'} + I_j^{V'})}{N_j} \tag{24}$$

β is the transmission rate of the first strain, σ indicates the relative transmissibility of the second strain compared to the previously circulating one. $\mathbf{C'}$ is the contact matrix adjusted for NPIs. To avoid issues with transition probabilities large than one, we transform the rates λ_k and $\lambda_k^{'}$ with the function $f(\lambda) = 1 - e^{-\lambda}$. Furthermore, $Mult_1(X, p_1, p_2)$ and $Mult_2(X, p_1, p_2)$ describe, respectively, a draw from the random variable 1 occurring with probability p_1 and the random variable 2 occurring with probability p_2, given X trials.

In the behavioural models we add non-compliant compartments. In *constant rate model* and *time-varying rate model* non vaccinated and vaccinated susceptible can both become non-compliant (transitioning to, respectively, S_{NC} and S_{NC}^V). In *constant rate model (vaccinated only)* and *time-varying rate model (vaccinated only)* the behavioural relaxation is linked only to vaccinated susceptible individuals. The transitions among compartments in *constant rate model* and *time-varying rate model* are as following:

$$\begin{aligned}
S_k(t+\delta t) &= S_k(t) - Mult_1(S_k(t), \lambda_k, \lambda_k', h(\alpha)) - Mult_2(S_k(t), \lambda_k, \lambda_k', h(\alpha)) \\
&\quad - Mult_3(S_k(t), \lambda_k, \lambda_k', h(\alpha)) + Mult_3(S_{NCk}(t), r\lambda_k, r\lambda_k', h(\gamma)) && (25) \\
S_{NCk}(t+\delta t) &= S_{NCk}(t) - Mult_1(S_{NCk}(t), r\lambda_k, r\lambda_k', h(\gamma)) - Mult_2(S_{NCk}(t), r\lambda_k, r\lambda_k', h(\gamma)) \\
&\quad - Mult_3(S_{NCk}(t), r\lambda_k, r\lambda_k', h(\gamma)) + Mult_3(S_k(t), \lambda_k, \lambda_k', h(\alpha)) && (26) \\
L_k(t+\delta t) &= L_k(t) + Mult_1(S_k(t), \lambda_k, \lambda_k', h(\alpha)) + Mult_1(S_{NCk}(t), r\lambda_k, r\lambda_k', h(\gamma)) \\
&\quad - Bin(L_k(t), \epsilon) && (27) \\
L_k'(t+\delta t) &= L_k'(t) + Mult_2(S_k(t), \lambda_k, \lambda_k', h(\alpha)) + Mult_2(S_{NCk}(t), r\lambda_k, r\lambda_k', h(\gamma)) \\
&\quad - Bin(L_k'(t), \epsilon) && (28) \\
S_k^V(t+\delta t) &= S_k^V(t) - Mult_1(S_k^V(t), (1-VE_S)\lambda_k, (1-VE_S')\lambda_k', h(\alpha)) \\
&\quad - Mult_2(S_k^V(t), (1-VE_S)\lambda_k, (1-VE_S')\lambda_k', h(\alpha)) \\
&\quad - Mult_3(S_k^V(t), (1-VE_S)\lambda_k, (1-VE_S')\lambda_k', h(\alpha)) \\
&\quad + Mult_3(S_{NCk}^V(t), r(1-VE_S)\lambda_k, r(1-VE_S')\lambda_k', h(\gamma)) && (29) \\
S_{NCk}^V(t+\delta t) &= S_{NCk}^V(t) - Mult_1(S_{NCk}^V(t), r(1-VE_S)\lambda_k, r(1-VE_S')\lambda_k', h(\gamma)) \\
&\quad - Mult_2(S_{NCk}^V(t), r(1-VE_S)\lambda_k, r(1-VE_S')\lambda_k', h(\gamma)) \\
&\quad - Mult_3(S_{NCk}^V(t), r(1-VE_S)\lambda_k, r(1-VE_S')\lambda_k', h(\gamma)) \\
&\quad + Mult_3(S_k^V(t), (1-VE_S)\lambda_k, (1-VE_S')\lambda_k', h(\alpha)) && (30) \\
L_k^V(t+\delta t) &= L_k^V(t) + Mult_1(S_k^V(t), (1-VE_S)\lambda_k, (1-VE_S')\lambda_k', h(\alpha)) \\
&\quad + Mult_1(S_{NCk}^V(t), r(1-VE_S)\lambda_k, r(1-VE_S')\lambda_k', h(\gamma)) - Bin(L_k^V(t), \epsilon) && (31) \\
L_k^{V'}(t+\delta t) &= L_k^{V'}(t) + Mult_2(S_k^V(t), (1-VE_S)\lambda_k, (1-VE_S')\lambda_k', h(\alpha)) \\
&\quad + Mult_2(S_{NCk}^V(t), r(1-VE_S)\lambda_k, r(1-VE_S')\lambda_k', h(\gamma)) - Bin(L_k^{V'}(t), \epsilon) && (32)
\end{aligned}$$

The transitions of the rest compartments are the same as those in the baseline model, described by Eqs 3-22.

Constant rate model (vaccinated only) and *time-varying rate model (vaccinated only)* can be described by Eqs. 29-32, 1-11, 14-17, and 19-22.

1.2 R_0 calculation

We calculate the basic reproductive number R_0 of proposed model using the next generation matrix approach [3]. By definition, R_0 is the reproductive number at the beginning of the epidemics. Although

our models include the emergence of a second variant, vaccines, and relaxation of individual behaviours, all of these become relevant only after the start of the epidemic. As such they have no influence on R_0. Thus, we can disregard the compartments related to the second variant, vaccination, and behavioural relaxation when we calculate R_0. We consider only the infected individuals in the compartments L_k and I_k. The deterministic equations regulating the dynamics of these two compartments are:

$$\frac{dL_k}{dt} = \lambda_k S_k - \epsilon L_k \tag{33}$$

$$\frac{dI_k}{dt} = \epsilon_k L_k - \mu I_k \tag{34}$$

We have $K = 16$ age groups, thus both Eqs. 33 and 34 contains K equations for different age groups. We describe these $2K$ equations in matrix form:

$$\begin{bmatrix} \frac{dL_1}{dt} \\ \vdots \\ \frac{dL_k}{dt} \\ \frac{dI_1}{dt} \\ \vdots \\ \frac{dI_k}{dt} \end{bmatrix} = \begin{bmatrix} \lambda_1 S_1 \\ \vdots \\ \lambda_k S_k \\ 0 \\ \vdots \\ 0 \end{bmatrix} - \begin{bmatrix} \epsilon L_1 \\ \vdots \\ \epsilon L_k \\ -\epsilon L_1 + \mu I_1 \\ \vdots \\ -\epsilon L_k + \mu I_k \end{bmatrix} \tag{35}$$

where $\lambda_k = \beta \sum_{j=1}^{N_k} \mathbf{C'}_{kj} I_j / N_j$ is the force of infection of the original strain, and the contact matrix $\mathbf{C'}$ accounts for the change in contacts induced by NPIs. We further denote Eq. 35 as

$$\begin{bmatrix} \frac{d\theta_1}{dt} \\ \vdots \\ \frac{d\theta_k}{dt} \\ \frac{d\theta_{k+1}}{dt} \\ \vdots \\ \frac{d\theta_{2k}}{dt} \end{bmatrix} = \begin{bmatrix} F_1 \\ \vdots \\ F_k \\ 0 \\ \vdots \\ 0 \end{bmatrix} - \begin{bmatrix} V_1 \\ \vdots \\ V_k \\ V_{k+1} \\ \vdots \\ V_{2k} \end{bmatrix} \tag{36}$$

For age group k, we consider the disease free equilibrium (DFE), defined as (S_k, L_k, I_k, R_k)=(N_k, 0, 0, 0). Next, we define two matrices: $\mathbf{F}_{ij}|_{DFE} = \frac{dF_i}{d\theta_j}$, $\mathbf{V}_{ij}|_{DFE} = \frac{dV_i}{d\theta_j}$. Considering the DFE, we write down $\mathbf{F}$ and $\mathbf{V}$ as follows.

$$F = \begin{bmatrix} 0 & \cdots & 0 & \frac{\beta N_1 C'_{1K} \chi}{N_K} & \cdots & \frac{\beta N_1 C'_{1K} \chi}{N_K} \\ \vdots & \ddots & \vdots & \vdots & \ddots & \vdots \\ 0 & \cdots & 0 & \frac{\beta N_K C'_{K1} \chi}{N_K} & \cdots & \frac{\beta N_K C'_{K1} \chi}{N_1} \\ 0 & \cdots & 0 & 0 & \cdots & 0 \\ \vdots & \ddots & \vdots & \vdots & \ddots & \vdots \\ 0 & \cdots & 0 & 0 & \cdots & 0 \end{bmatrix} \tag{37}$$

$$V = \begin{bmatrix} \epsilon & \cdots & 0 & 0 & \cdots & 0 \\ \vdots & \ddots & \vdots & \vdots & \ddots & \vdots \\ 0 & \cdots & \epsilon & 0 & \cdots & 0 \\ -\epsilon & \cdots & 0 & \mu & \cdots & 0 \\ \vdots & \ddots & \vdots & \vdots & \ddots & \vdots \\ 0 & \cdots & -\epsilon & 0 & \cdots & \mu \end{bmatrix} \tag{38}$$

The reproductive number R_0 is defined as $\rho(\mathbf{FV^{-1}})$, where $\rho(\cdot)$ represents the spectral radius. Then, we write $\mathbf{F}$ and $\mathbf{V}^{-1}$ in blocks and we compute $\mathbf{V^{-1}}$ as follows:

$$\mathbf{F} = \begin{bmatrix} 0 & \beta\tilde{\mathbf{C}}' \\ 0 & 0 \end{bmatrix} \tag{39}$$

where $\tilde{\mathbf{C}}'$ indicates the adjusted contact matrix ($\tilde{\mathbf{C}}'_{ij} = \mathbf{C}'_{ij}\frac{N_i}{N_j}$) with a size of $K \times K$, and 0 indicates a $K \times K$ matrix with all zero elements.

$$\mathbf{V} = \begin{bmatrix} \epsilon\mathbb{1} & 0 \\ -\epsilon\mathbb{1} & \mu\mathbb{1} \end{bmatrix} \tag{40}$$

where $\mathbb{1}$ indicates a $K \times K$ identity matrix. Then we compute $\mathbf{V^{-1}}$

$$\mathbf{V^{-1}} = \begin{bmatrix} \frac{1}{\epsilon}\mathbb{1} & 0 \\ \frac{1}{\mu}\mathbb{1} & \frac{1}{\mu}\mathbb{1} \end{bmatrix} \tag{41}$$

Then, we obtain $\mathbf{FV^{-1}}$

$$\mathbf{FV^{-1}} = \begin{bmatrix} 0 & \beta\tilde{\mathbf{C}}' \\ 0 & 0 \end{bmatrix} \begin{bmatrix} \frac{1}{\epsilon}\mathbb{1} & 0 \\ \frac{1}{\mu}\mathbb{1} & \frac{1}{\mu}\mathbb{1} \end{bmatrix} = \begin{bmatrix} \frac{\beta\tilde{C}'}{\mu} & \frac{\beta\tilde{C}'}{\mu} \\ 0 & 0 \end{bmatrix} \tag{42}$$

Finally, we are left with finding the spectral radius of $\mathbf{FV^{-1}}$ (i.e., finding its largest eigenvalue). The eigenvalue problem can be written as $det(\mathbf{FV^{-1}} - \lambda\mathbb{1}) = 0$. Given the structure of $\mathbf{FV^{-1}}$, and since we are interested in non-trivial solutions (λ not equal to 0), the problem reduces to:

$$det(\frac{\beta\tilde{\mathbf{C}}'}{\mu} - \lambda\mathbb{1}) = 0 \tag{43}$$

Therefore, we obtain $R_0 = \rho(\mathbf{FV^{-1}}) = \frac{\beta}{\mu}\rho(\tilde{\mathbf{C}}')$.

1.3 Sensitivity to the modelling framework

In the main text, we adopted a simple SLIR compartmentalization that does not account for presymptomatic and asymptomatic individuals. While similar approaches have been used to model COVID-19 in the literature [4–6], we acknowledge the simplicity of this compartmentalization setup. As a sensitivity check, here we consider a more detailed compartmental structure: the Susceptible-Latent-Presymptomatic-Asymptomatic/Infected-Recovered (SLPAIR) model. In detail, we add two new compartments, presymptomatic (P) and asymptomatic (A). In this new framework the epidemic progresses as follows. Susceptible (S) individuals transit to latent stage (L) by interacting with individuals that can transmit the disease, namely those in compartments P, A, and I. Latent individuals transit to the presymptomatic phase (P) after the latent period ϵ^{-1}. Then, at a rate ω, P individuals become either asymptomatic (A) with a probability f (here set to 0.2 according to Ref. [7]) or symptomatic (I) with probability $(1-f)$. The time spent in compartment L (ϵ^{-1}) added to the time spent in compartment P (ω^{-1}) yields the incubation period. Infectiousness of individuals in compartments P and A is reduced by a factor χ (here set to 0.67 according to Ref. [8, 9]). Therefore, the force of infection for age group k is given by:

$$\lambda_k = \beta \sum_{j=1}^{K} \frac{\mathbf{C}'_{kj}[\chi(P_j + P_j^V + A_j + A_j^V) + I_j + I_j^V]}{N_j} \tag{44}$$

We assume that asymptomatic individuals (A) do not develop severe symptoms and eventually recover after the infectious period μ^{-1}. Symptomatic individuals (I), after the infectious period μ, either recover or die according to the age-stratified infection fatality rate. Analogous to what we did in the main text for the SLIR model, we duplicate the compartments to capture the emergence of a second virus strain. For simplicity, we keep the time spent in the presymptomatic phase the same for all strains considered. The

transmission rate of the second strain is increased by a factor σ, similar to what we did for SLIR models in the main text. The force of infection related to the second strain is then:

$$\lambda'_k = \beta\sigma \sum_{j=1}^{K} \frac{\mathbf{C'}_{kj}[\chi(P'_j + P^{V'}_j + A'_j + A^{V'}_j) + I'_j + I^{V'}_j]}{N_j} \tag{45}$$

We also double the compartments to incorporate the vaccinated population. The vaccine has an efficacy VE_S (or VE'_S for the second strain) against infection and an efficacy VE_M (or VE'_M for a second strain) against death. The compartmental structure of the SLPAIR is shown in Fig. A.

We use the same ABC-SMC algorithm to calibrate the SLPAIR models. In Fig. B we show the calibrated models and the real epidemic data. The fits are similar to those obtained for SLIR models, except for the simulated second peak for São Paulo, which is a lower than the observed data. To evaluate the models' performance, we compute the wMAPEs (see Tab. A), AIC weights and BIC weights (see Tab B) as done for SLIR models. The SLPAIR models show slightly better fit than SLIR models in British Columbia, Lombardy, and London, but a worse fit in São Paulo. In line with the results of SLIR models, SLPAIR models with behavioural component feature improved performance with respect to the baseline as evidenced by lower wMAPEs. We find a decrease in wMAPE of 2% in British Columbia, 23% in Lombardy, 4% in London, and 7% in São Paulo with respect to the baseline. As discussed in the main text, the improvements in case of a SLIR compartmentalization are 10% in Lombardy, 2% in London, and 6% in São Paulo compared to the baseline. These numbers are in line, though the SLPAIR models report more pronounced reductions in the wMAPEs generally. However, as mentioned above, we note that for São Paulo all models (including the baseline) miss to capture the extent of the second peak.

In terms of AIC weights, the results obtained for SLPAIR models are consistent with those shown in Tab. 3 of the main text for SLIR models. In both frameworks, only one behavioural model, the *constant rate model* shows the highest AIC weight in Lombardy, while the baseline model reports the highest AIC weight in the other three regions. The results of BIC weights are also similar in the two settings with one notable exception in the *constant rate model* in Lombardy. Under the SLPAIR framework, this model shows the highest BIC weight in Lombardy, in line with the AIC ranking. When considering the SLIR framework, the model with the highest BIC in Lombardy is instead the baseline.

In general, the picture emerging considering a SLPAIR compartmentalization is largely consistent with what discussed in the main text for SLIR models. The behavioural mechanisms improve the fit as evidenced by lower wMAPEs. However, the increased complexity offsets the improvement of fit in the three out of regions studied.

Table A. wMAPEs obtained comparing the medians of calibrated SLPAIR models and reported weekly deaths. The lowest wMAPE in each location, indicating best performance, is highlighted in bold.

wMAMPE	Baseline	Constant rate	Time-varying rate	Constant rate (vaccinated only)	Time-varying rate (vaccinated only)
British Columbia	0.324	0.338	0.332	**0.319**	0.321
Lombardy	0.286	**0.221**	0.281	0.285	0.302
London	0.151	**0.145**	0.148	0.149	0.147
São Paulo	0.364	0.351	**0.337**	0.341	0.357

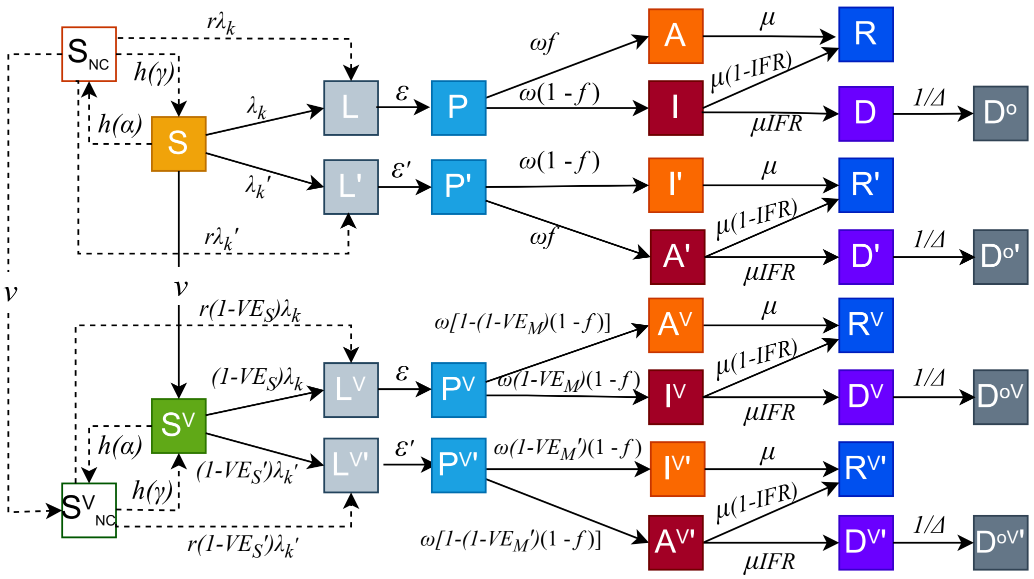

Fig A. Compartmental structure for SLPAIR model. All compartments connected by solid lines constitute the baseline model. This baseline model includes susceptible (S), latent (L), presymptomatic (P), asymptomatic (A), infected (I), recovered (R), and dead (D, D^o) compartments. The top row represents non-vaccinated compartments, whereas the bottom row represents vaccinated compartments. Individuals in the S compartment get vaccinated according to real vaccine rates (ν) and then transition to the S^V compartment. To account for the emergence of a second strain, we double the compartments creating L', I', R', D', and $D^{o'}$. This is done also for the vaccinated compartments that become $L^{V'}$, $I^{V'}$, $R^{V'}$, $D^{V'}$, $D^{Vo'}$. Behavioural models include susceptible non-compliant compartments (S_{NC}, S^V_{NC}) connected by dotted lines, where individuals have r times higher probability of getting infected with respect to susceptible compliant individuals (S and S^V). In the *constant rate model* and the *time-varying rate model*, we include S_{NC} and S^V_{NC}, whereas in the vaccinated-only versions, we include only S^V_{NC}.

Table B. AIC and BIC weights computed considering calibrated SLPAIR models' medians and reported weekly deaths. The highest AIC/BIC weight in each location is highlighted in bold.

AIC	Baseline	Constant rate	Time-varying rate	Constant rate (vaccinated only)	Time-varying rate (vaccinated only)
British Columbia	**0.82**	0.04	0.02	0.07	0.05
Lombardy	0.00	**1.00**	0.00	0.00	0.00
London	**0.77**	0.08	0.06	0.03	0.06
São Paulo	**0.46**	0.06	0.23	0.20	0.05
BIC					
British Columbia	**0.98**	0.00	0.00	0.01	0.01
Lombardy	0.01	**0.99**	0.00	0.00	0.00
London	**0.97**	0.01	0.01	0.00	0.01
São Paulo	**0.90**	0.01	0.04	0.04	0.01

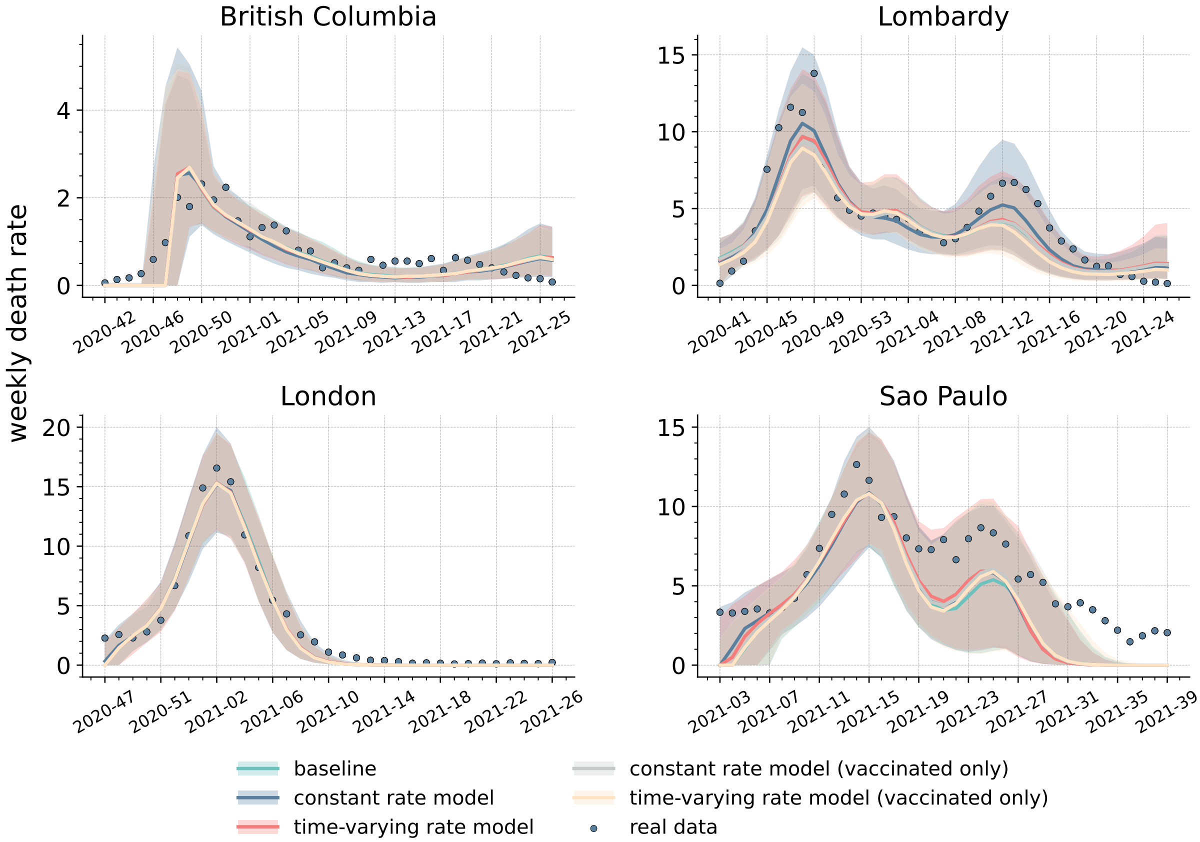

Fig B. Comparison of baseline and behavioural models under the SLPAIR compartmentalization. Calibrated weekly deaths trajectories (i.e., weekly deaths per $100,000$) for the baseline and four behavioural models across the four regions. Solid lines indicate the medians, while the shaded areas the 90% confidence intervals. Reported weekly deaths are denoted by blue dots.

2 Demographics of regions considered

Here, we provide more information about the demographic profiles of the four regions under study.

2.1 Contact matrices

We first present the contact patterns among different age groups. In Fig. C, we show the pre-Pandemic contact matrices of each region in four settings (i.e., home, school, work places, and other places) sourced from Ref. [2]. Across settings and regions we use the same colour scale for comparison. The plot shows that, across the board, the four regions exhibit similar contact patterns. In detail, the contacts at home show a higher intensity along the diagonal. Contacts at school are the most intense compared to the other three settings, where interactions are mainly among children and adolescents (i.e., age brackets $0-4$, $5-94$, $10-14$, $15-19$). This is followed by contacts at other places among the young population (i.e., $10-14$, $15-19$, $20-24$, $25-29$). Contacts at workplaces are less intense than in schools and other places, and are reported mainly among the middle-aged population. By considering contacts across all four settings, we obtain the aggregated contact matrices shown in the last column. The darker colour of the diagonal suggests that within-group interactions (i.e., among people in the same age group) are significantly more frequent than interactions across different age groups. Additionally, teenagers and young adults tend to have more contacts than the elderly population. To compare the contact matrices of the four regions, we compute the spectral radius of the aggregated contact matrix. We find that the spectral radius is 15.2 in British Columbia, 17.0 in Lombardy, 11.7 in London, and 19.5 in São Paulo. As discussed in the previous section, this indicates that, in the context of epidemic spreading, for a given disease London would feature the lowest R_0 while São Paulo the largest.

2.2 Spectral radius of contact matrices as a function of time

We use several datasets to model the impact of NPIs on contact patterns. Specifically, we compute contact reduction factors using mobility and policy data (see Eq. 2 in the main text). To visualize the modulation in contact intensity due to NPIs, here we show the spectral radius of contact matrices over time in the four regions. The spectral radius, defined as the largest eigenvalue, directly contributes to the reproductive number of the model, and thus is often considered as a proxy for intensity of contacts relevant to the spreading. For easier interpretation of temporal trends, we compute the ratio between the spectral radius of the contact matrices at time t, denoted by $\rho(\mathbf{C}'_t)$, and the values on the first simulation day, denoted by $\rho(\mathbf{C}'_0)$. As shown in Fig. D, in Lombardy, the ratio drops to below 0.6 around week 47 of 2020 and fluctuates until week 12 of 2021, indicating the most pronounced and sustained relative decrease in contacts among the four regions. In London, the ratio suddenly drops to around 0.6 in week 53 of 2020 and stays at a stable level until week 10 of 2021, then returning to the level on the first day of the simulations. In British Columbia, the ratio fluctuates around 1, with a small drop in week 53 of 2020. In São Paulo, the ratio drops later compared to the other regions (as this region experienced epidemic waves later), in week 10 and achieves the lowest value during week $13-14$ of 2021. Then, it quickly increases and even surpasses 1 after week 15 of 2021. These results are consistent with the mobility levels over time shown in Fig. 1B in the main text. We note however how in that figure we opted to show a different, but related, indicator to capture the changes in contact patterns. Indeed, we showed the average modulation of the contact matrices considering various contexts (i.e., work, school, others) as estimated from data.

2.3 Contact intensity and population distribution

In Fig. E we show the pre-Pandemic contact intensity (panel A) and age distribution (panel B) for each age group in the four regions. Contact intensity is computed by summing each column of the overall contact matrix.

Younger age groups, particularly children and adolescents, exhibit the highest contact intensity across all regions, while the elderly population (i.e., 65+) the lowest. In detail, contact intensity peaks in the $15-19$ age group, followed by a general decline with age with small fluctuations including a slight increase

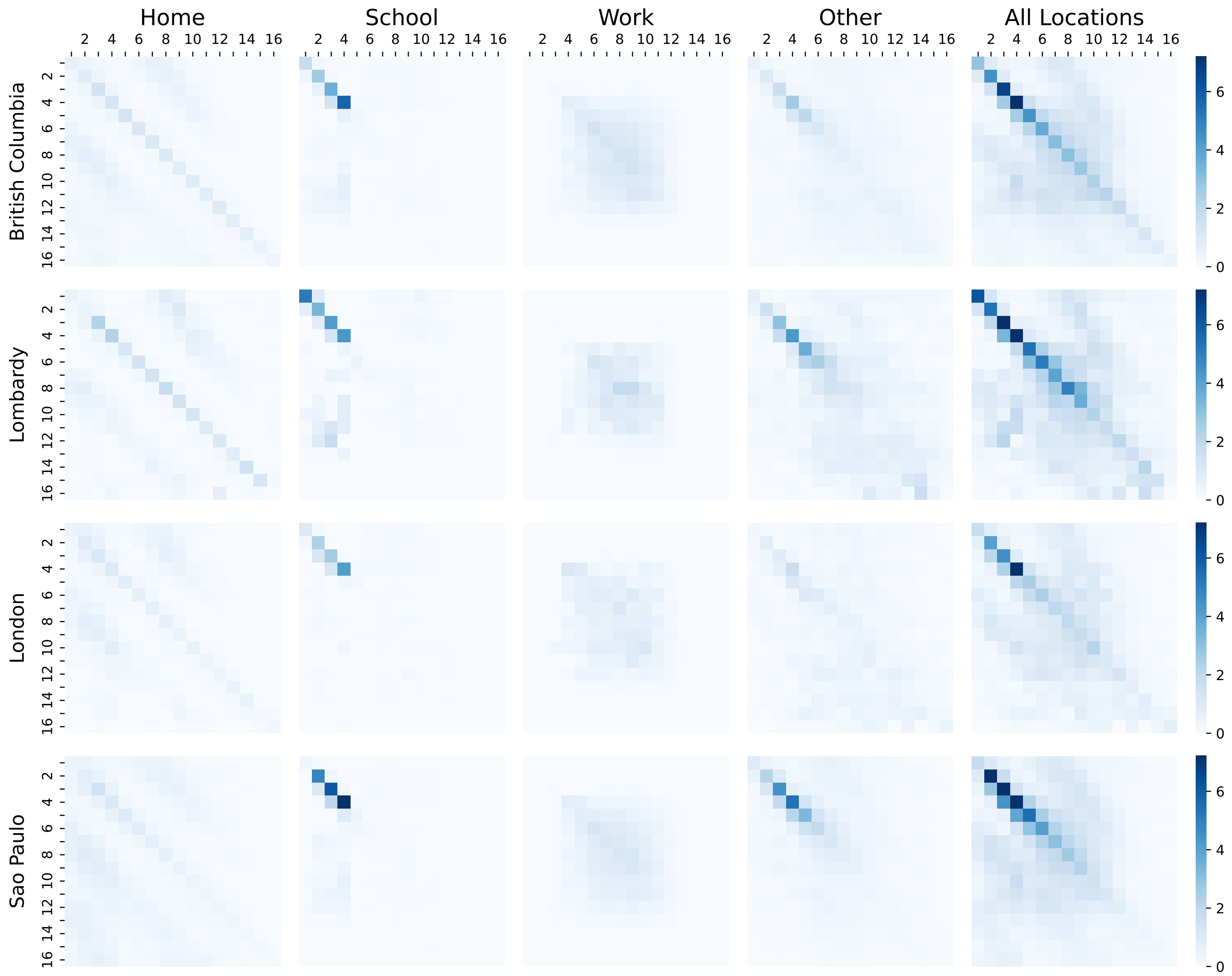

Fig C. Contact matrices of the four regions. We show the four layers (i.e., home, school, workplace, and other places) of contact matrices between 16 age groups in each region (British Columbia, Lombardy, London, São Paulo). The numbers shown on the top and left of the matrices indicates age groups (e.g., 2 represents age group $5-9$ and 16 represents age group 75+). The last column shows aggregated contact matrices considering all interaction settings.

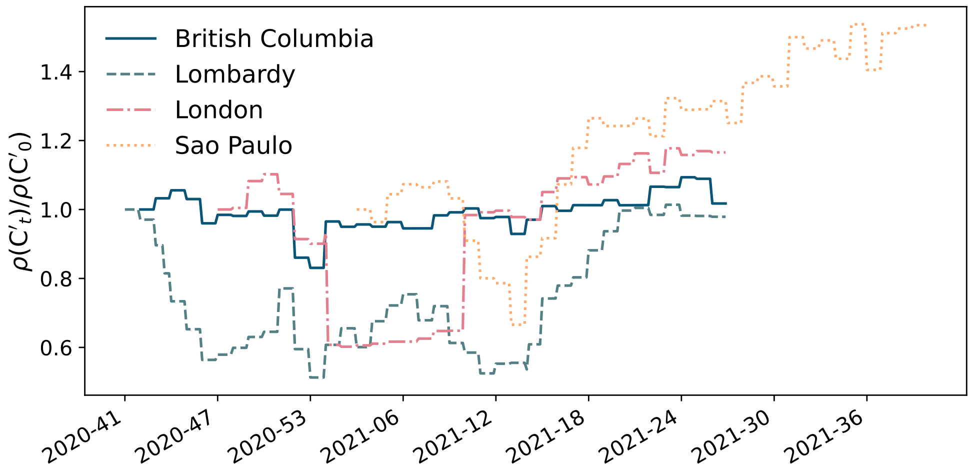

Fig D. Spectral radius of contact matrices over time. We show the ratio of the spectral radius of contact matrix at time t with respect to the value at the beginning of the simulation period in each region. The start and end dates correspond to the observed time of each region in our study.

in the $35-39$ age group (except in São Paulo, where the decline is consistent). A downward trend in contact intensity is observed from the $40-44$ age group onwards in all regions.

Fig. E-B shows the population distributions of the four regions in 2021 [10–13]. In British Columbia, the population is relatively evenly distributed across most age groups, with small double peaks in the $30-34$ to $55-59$ age groups. Lombardy shows a gradual increase in population from the $30-34$ age group to the $50-54$ age group. The population distribution in London peaks in the $30-34$ age group, suggesting a substantial proportion of young adults. São Paulo shows a peak in the $35-39$ age group. Comparing the population distributions of the four regions, British Columbia and Lombardy display signs of ageing and also display smaller populations in younger age categories (i.e., $0-4$, $5-9$ age groups).

2.4 Validation of contacts reduction estimation: comparison with independent data from Lombardy

In this sub-section, we compare our estimates regarding the modulation of contacts induced by NPIs with empirical data collected via surveys in Ref. [14]. The authors report reductions of 16%, 30%, and 42% in daily contacts under mild, moderate, and strong restrictions from October 26, 2020 to March 31, 2021. These reductions are computed with respect to a baseline period from July 10, 2020 to October 25, 2020 defined prior the implementation of a NPIs tier system in Italy. To compare the modulation in contacts, we calculate the ratio between the number of contacts in our NPI-adjusted contact matrices during the same period and the average number of contacts in the same baseline period. The ratio varies from 0.63 to 1.0, i.e., a maximum reduction of 37% in contacts with respect to the baseline level. These values are largely in line with the results reported in Ref. [14]. Besides, they compared their results with the Google Mobility Reports finding a positive correlation of 0.30, considering contacts in workplaces, 0.87 in retail and recreation, and 0.80 in transit stations. Overall, these results provide independent support for our methodology.

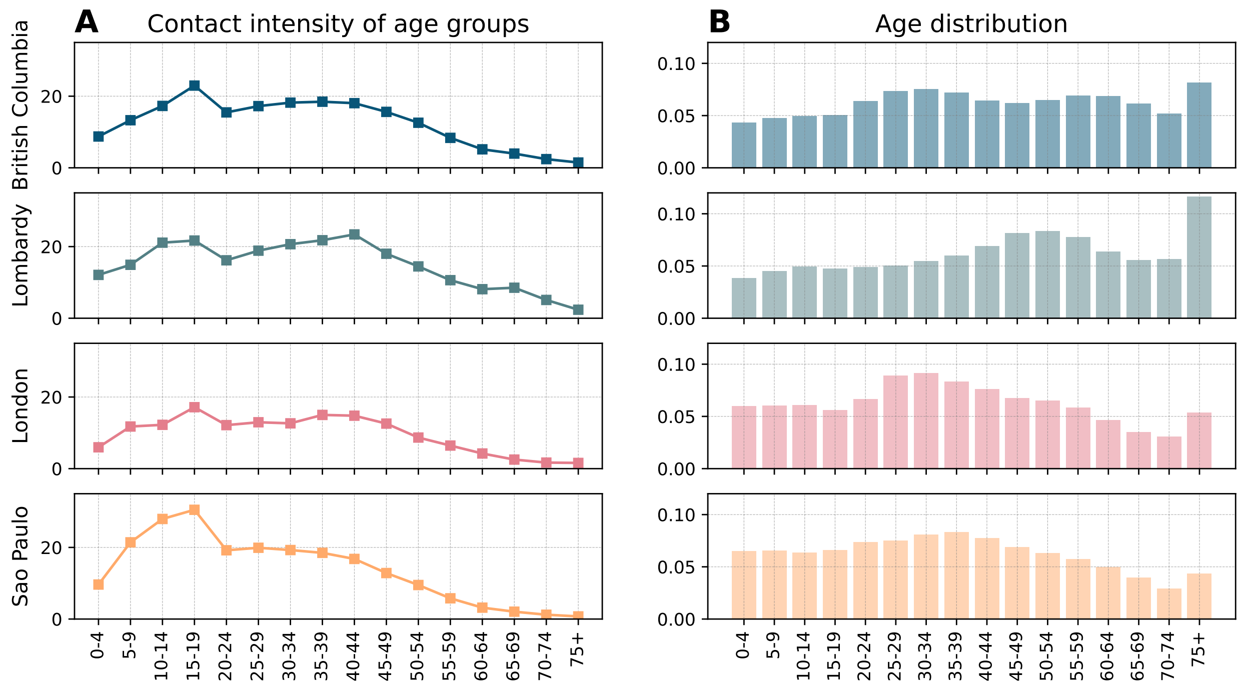

Fig E. Contact intensity and demographics. A) Contact intensity across age groups in the four regions. Contact intensity is computed by summing each column of the contact matrix. B) Age distributions across the four regions with a 5-year bracket except for the last 75+ group.

3 Impact of vaccines and NPIs

3.1 Timing of vaccination impact

In the context of the no-vaccination counterfactual with the baseline model, we investigate when vaccinations start to have a macroscopic impact. To this end, we compute the time when the weekly deaths with/without vaccinations begin to diverge by at least 1% (i.e., the weekly relative difference of deaths exceeds this threshold). The results are shown in Fig. F. The grey lines mark the start of vaccinations, and the red lines denote the week in which the vaccination begins to have impact. We find that the vaccination impact started 18 weeks after the first rollout in British Columbia, 6 weeks in Lombardy, 8 weeks in both London and São Paulo. British Columbia experienced a much lower epidemic burden, thus the macroscopic impact of vaccination shows later than the other regions. Notably, the weekly RDD drops to 0 after 2021-15 in London as there are no deaths (the denominator becomes 0).

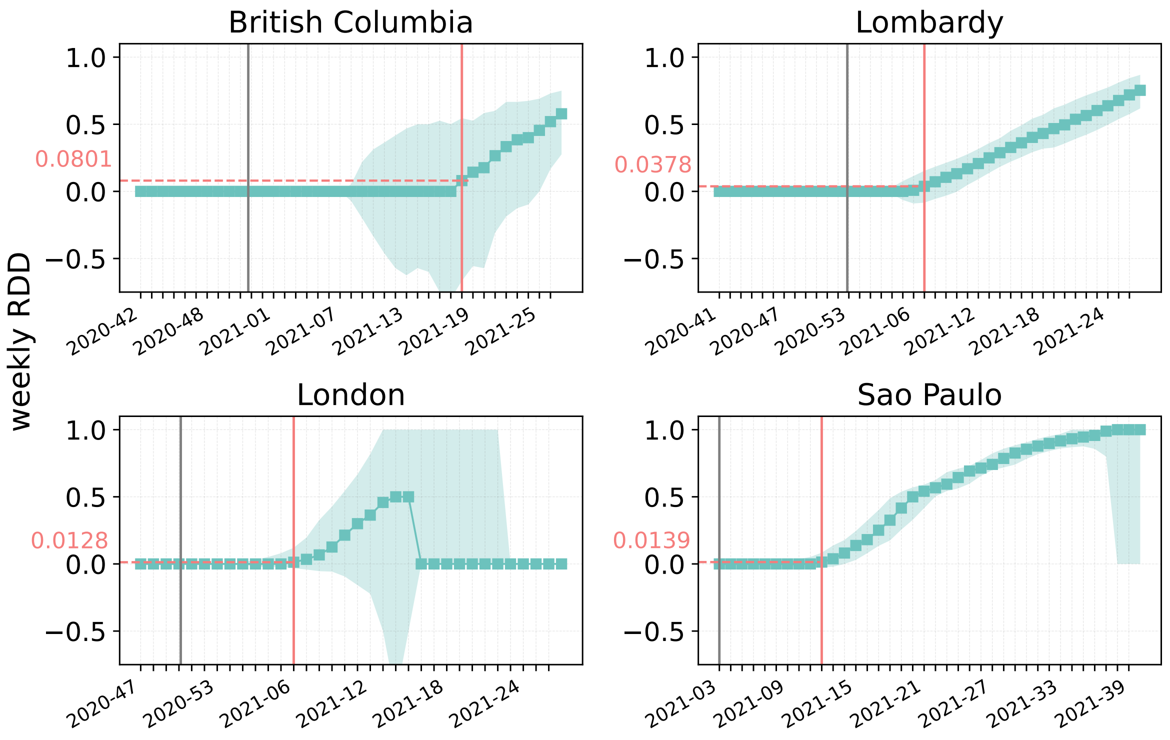

Fig F. Weekly relative deaths difference in counterfactual scenarios without vaccinations. The fraction of deaths averted by vaccination each week with respect to an equivalent model without vaccinations. The grey lines mark the start of vaccinations while the red lines mark the week in which the relative difference of deaths is larger than 0.01 for the first time (0.0801 in British Columbia, 0.0079 in Lombardy, 0.0128 in London, 0.0139 in São Paulo). We show the results considering 1000 stochastic trajectories, median and 90% confidence interval.

3.2 Influence of NPIs on peak deaths

We analyze the impact of non-pharmaceutical interventions (NPIs) on deaths peak by calculating the fold increase in the peak intensity in a scenario without NPIs compared to the original model with NPIs. In doing so, we maintain vaccination in both scenarios. We first show the death trajectories with/without NPIs in Fig. G. The peak of deaths without NPIs (red lines) is much higher than with NPIs (blue lines). The peak without NPIs also comes earlier (in terms of the median trajectory) compared to those with NPIs in Lombardy and London (3 weeks earlier) and São Paulo (5 weeks earlier). Next we calculate the fold increase in peak deaths of 1000 stochastic trajectories in Fig. H. Removing NPIs would have resulted in 5.3 (90% CI: [3.3, 9.4]) times higher in British Columbia, 8.8 [6.8, 11.9] times higher in Lombardy, 6.7 [5.5, 8.2] times higher in London, and 4.7 [3.9, 5.7] times higher in São Paulo times higher peaks. São Paulo reports the lowest fold in peak deaths as it reports the least stringent NPIs compared to the other three regions. Lombardy reports the highest fold in peak death, though London adopted the most stringent NPIs. This may be due to a demographic factor. Indeed, Lombardy has a larger proportion of senior population, associated with a higher infection fatality rate due to increased vulnerability to severe outcomes.

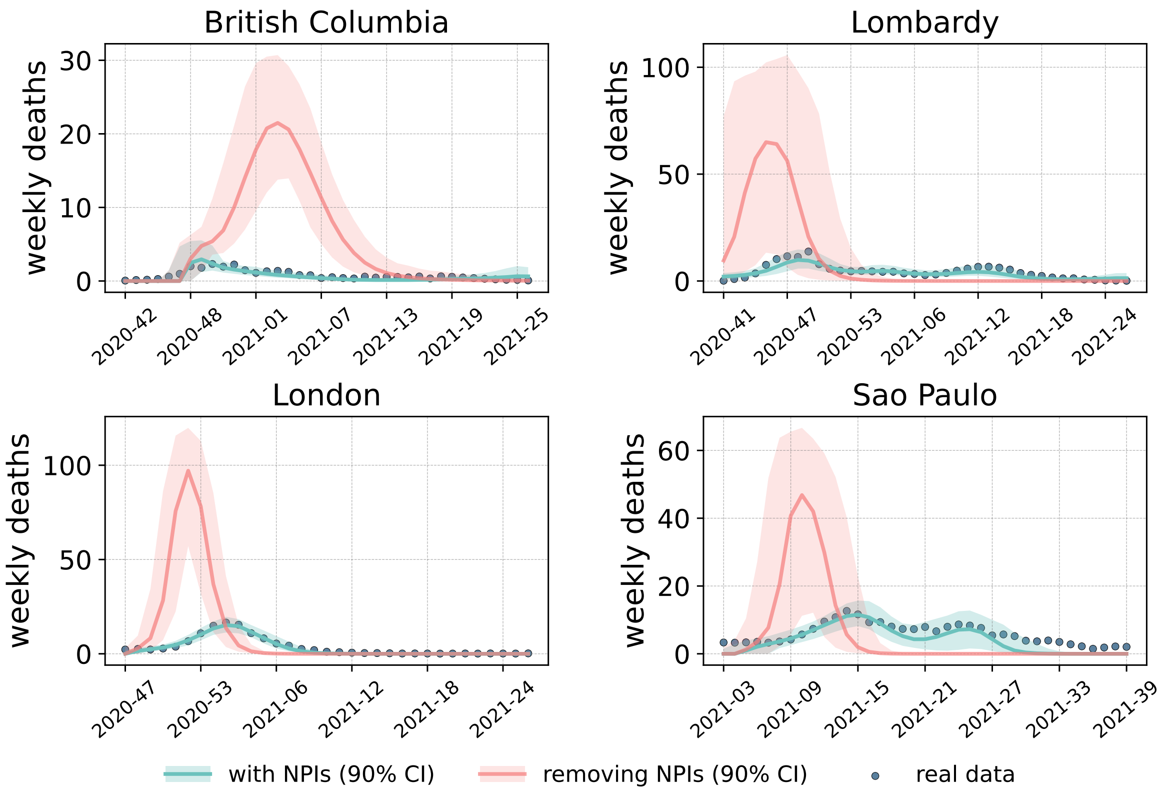

Fig G. Comparison of weekly death trajectories with/without NPIs. Calibrated weekly death trajectories (weekly deaths per $100,000$) of the baseline model (denoted by blue lines) and in a counterfactual scenario where NPIs are removed (denoted by red lines).

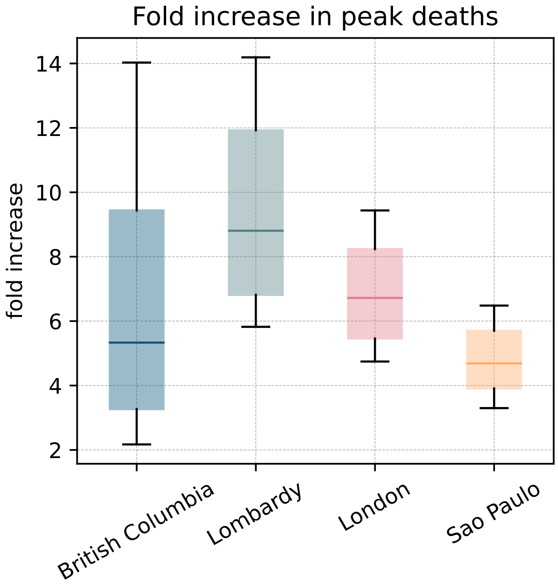

Fig H. Fold increase in peak deaths intensity in counterfactual scenarios without NPIs. The increase fold of peak deaths in a scenario where NPIs are removed with respect to an equivalent case with NPIs. The box plots show the results of averted deaths considering 1000 stochastic trajectories in each region.

3.3 Relative difference of infection in counterfactual scenarios without vaccination or NPIs

In the main text, we presented the relative deaths difference (i.e., RDD) with/without vaccination, NPIs, and behavioural relaxation. Here, we show the analogous results for the relative difference in the number of infected (i.e., RDI). This quantity is defined as:

$$RDI = \frac{I_{counterfactual} - I_{original}}{I_{counterfactual}} \times 100\% \tag{46}$$

where $I_{original}$ and $I_{counterfactual}$ are the total number of simulated infections in the original model and in the counterfactual scenario respectively.

For example, in the case of NPIs $I_{counterfactual}$ is computed considering estimates from a model without them. Instead, $I_{original}$ is the corresponding value in matched model with NPIs.

The RDIs in a scenario without vaccination are shown in Fig. I-A. We compute the median of RDI with a 90% confidence interval considering 1000 stochastic trajectories. The results indicate that vaccination prevented 48.96% (90% CI: [33.69%,58.24%]) of infections in British Columbia, 27.5% ([17.65%,35.68%]) in Lombardy, 0.24% ([0.05%,1.03%]) in London, and 8.59% ([7.14%,13.89%]) in São Paulo. The figures show a similar pattern with the RDDs (shown in the main text) in British Columbia, Lombardy, and London. However, São Paulo shows a different pattern. Indeed, São Paulo has the largest vaccine coverage among the regions and it shows the highest RDD. However, it features a lower RDIs compared to RDDs. São Paulo experienced the Gamma and Delta variants, which reduce the vaccine efficacy against infections significantly (0.65 of Gamma variant and 0.6 of Delta variant).

The RDIs of a counterfactual scenario without NPIs are shown in Fig. I-B. We find 80.46% (90% CI: [67.79%,88.84%]) infections avoided by NPIs in British Columbia, 57.34% ([53.12%,61.83%]) in Lombardy, 50.85% ([43.76%,59.01%]) London in London, and 0.63% ($[-5.04\%, 10.09\%]$) in São Paulo. The RDIs of the four regions show similar patterns to the observations for RDDs except for London reporting slightly lower RDIs than Lombardy. This may be explained by a larger population of younger population in London with respect to Lombardy. Besides, by comparing panel A and panel B in Fig. I, we can conclude that overall, in the first months of vaccines rollout, NPIs averted more infections compared to those prevented by vaccinations. These results highlight one more time the importance of NPIs during the complex initial phases of the vaccination campaign.

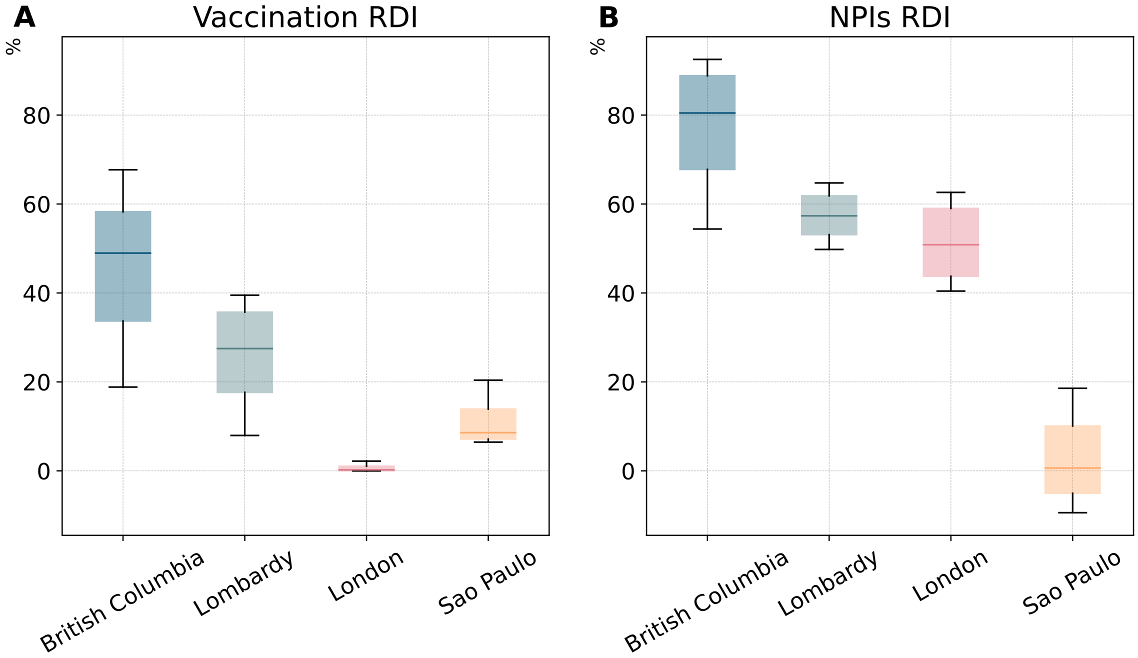

Fig I. Relative infection difference in counterfactual scenarios without vaccinations and NPIs. Panel A shows the fraction of total infections averted by vaccinations. Panel B shows the fraction of total infections averted by NPIs. The box plots show the results of averted deaths in 1000 stochastic trajectories in each region. The horizontal line within each box marks the median value, while the top and bottom edges correspond to the 0.95 and 0.05 quantiles (90% confidence interval). The whiskers extend to the maximum and minimum values. These estimates are obtained considering the baseline model.

4 Impact of behavioural relaxation

4.1 Behavioural transition rate

In *constant rate model* and 3 the transitions towards non-compliance and those back to compliance happen at constant rates, α and γ respectively. Instead, in models 2 and 4 these transitions are proportional to the fraction of vaccinated (multiplied by α) and deaths per $100,000$ (multiplied by γ). To better understand these varying transition rates, in Fig. J-A we plot, for different values of α, the transition rates from compliant to non-compliant compartments as a function of the fraction of vaccinated individuals. We denote this rate as $h(\alpha, frac_V(t)) = 1 - e^{-\alpha frac_V(t)}$, where $frac_V(t)$ is the fraction of vaccinated individuals of the total population. Similarly, in Fig. J-B we consider a range of γ values and plot the transition rates from compliant back to non-compliant as a function of daily new deaths per $100,000$ denoted by $h(\gamma, rate_D(t)) = 1 - e^{-\gamma rate_D(t)}$, where $rate_D(t)$ represents daily death rate (deaths per $100,000$).

The results in Fig. J-A show that a higher fraction of vaccinated individuals leads to an increased transition rates towards non-compliance. As α increases, the transition rate $h(\alpha, frac_V)$ rises more sharply. This means that, for a given fraction of vaccinated individuals, higher values of α result in a greater shift towards non-compliance. For example, when $\alpha = 0.1$, the transition rate is only 0.1 even when the fraction of vaccinated individuals reaches its maximum (1). In contrast, when $\alpha = 10$, the transition rate reaches 1 when only 40% of the population is vaccinated. A similar trend is observed in Fig. J-B where higher daily death rates lead to higher transition rates from non-compliance back to compliance. Furthermore, higher values of γ result in stronger responses to the epidemic's severity. For example, when $\gamma = 0.1$, the transition rate is 0.2 even when the daily death rate is quite high (2). In contrast, when $\gamma = 10$, the transition rate reaches the maximum when the death rate is smaller (0.5).

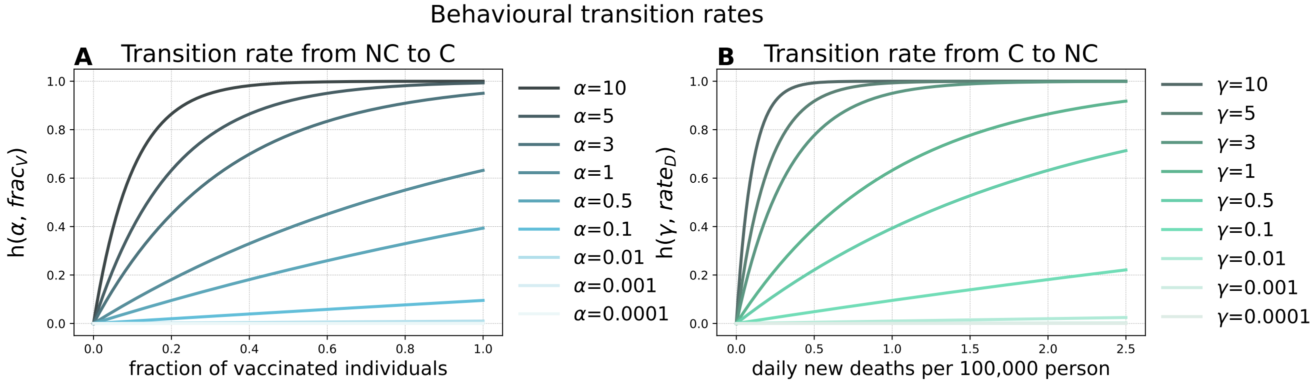

Fig J. Behavioural transition rates for models 2 and 4. In panel A we plot the behavioural transition rate from non-compliant to compliant denoted by $h(\alpha, frac_V)$ as a function of the fraction of vaccinated individuals for different values of α. In panel B we plot the behavioural transition rate from compliant to non-compliant denoted by $h(\gamma, rate_D(t))$ as a function of the fraction of daily new deaths per $100,000$ for different values of γ.

4.2 Fraction of non-compliant individuals as function of time

To have a more intuitive understanding on the four behavioural mechanisms, in Fig. K we plot the fraction of non-compliant individuals as function of time for the four models. To this end, we consider 1000 simulations obtaining sampling the posterior distribution of each model. We then compute the fraction of non-compliant individuals for each. We show the median trajectory with 90% confidence intervals. Overall, we observe that *constant rate model* result in the largest fraction of non-compliant individuals in the Lombardy and São Paulo. We also note that the *constant rate model* and *time-varying rate model* show the largest variability in terms of their confidence intervals. Besides, in the case of São Paulo, the median of

trajectory of the fraction of NC of *constant rate model* quickly raises to about 0.4 of the population, being stable for a while then showing a slow decrease. As shown in the main text, this is consistent with the large RDD by behavioural relaxation for this model and location.

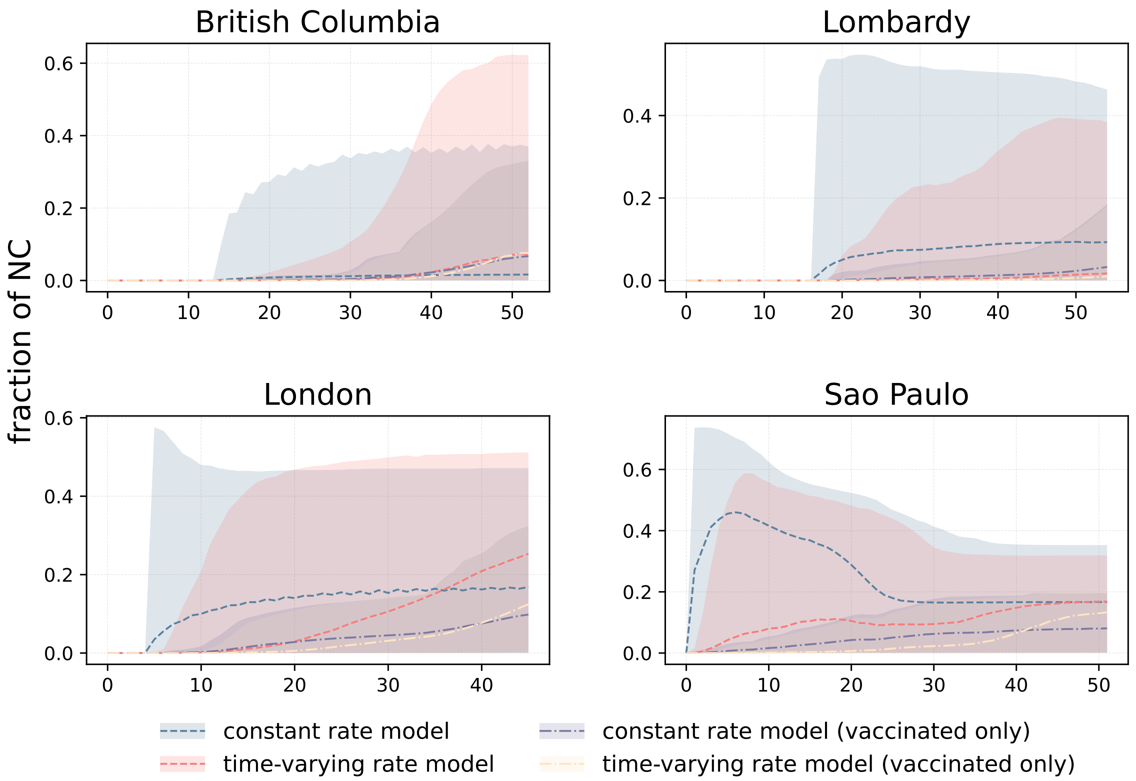

Fig K. Fraction of non-compliant individuals as function of time. We plot the fraction of non-compliant individuals of the four behavioural models in each region. The fractions are computed considering 1000 sampled trajectories. The dotted lines represent the median fractions, with shaded areas indicating the 90% confidence intervals.

4.3 Relative difference of infection in counterfactual scenarios without behavioural relaxation

We also calculate the relative difference of infections (RDIs) considering a counterfactual scenario where we remove the relaxation from the four behavioural models. The results are shown in Fig. L and Table C, displaying the median of RDIs along with 50% CIs. The values of RDI are below 0 in most cases, which means removing behavioural mechanisms leads to fewer infections (see Table C). The results for RDIs are consistent with the analogous for RDDs shown in the main text.

Table C. Relative infection difference in counterfactual scenarios without behavioural relaxation.

RDI (%)	Constant rate	Time-varying rate	Constant rate (vaccinated only)	Time-varying rate (vaccinated only)
British Columbia	-0.81 [-11.85,6.93]	-3.04 [-13.47,3.19]	-0.3 [-7.13,6.16]	-0.27 [-4.64,3.41]
Lombardy	-5.18 [-19.08,-0.86]	-0.78 [-2.96,0.0]	-0.52 [-1.38,0.04]	-0.27 [-0.91,0.21]
London	-1.39 [-7.36,-0.17]	-0.04 [-0.25,0.01]	-0.0 [-0.05,0.05]	-0.0 [-0.02,0.02]
São Paulo	-2.08 [-7.44,-0.12]	-1.54 [-7.76,-0.01]	-0.46 [-1.42,-0.08]	-0.15 [-0.83,-0.03]

4.4 Absolute death differences across counterfactual scenarios

In the main text, we showed the relative deaths difference considering counterfactual scenarios without vaccinations, NPIs, or behavioural relaxation. Here, we show absolute values. We show the medians with 90% confidence interval in Tables D-F.

As is shown in Table D, the absolute deaths difference in counterfactual scenarios without vaccination is small in British Columbia and London with a median of 144 and 114 more deaths without vaccinations than that with vaccination. In contrast, São Paulo shows a large difference with a median of more than $70K$.

Table D. Deaths difference in a counterfactual without vaccinations.

British Columbia	Lombardy	London	São Paulo
144 [30,544]	2988 [1682,4995]	114 [-6,418]	74376 [60593,88356]

The absolute deaths difference in counterfactual scenarios without NPIs is shown in Table E. The values are much higher than those of vaccination except a decrease in São Paulo. As mentioned in the main text, in São Paulo we observed the least stringent adoption of NPIs. These results underscore the importance of vaccinations, especially in settings with low adoption of NPIs.

British Columbia	Lombardy	London	São Paulo
9266 [7515,11633]	31337 [26775,37965]	22256 [18296,27550]	39448 [30069,54683]

Table E. Deaths difference in a counterfactual without NPIs

In Table F, we show the deaths difference in counterfactual scenarios without behavioural relaxation. *Constant rate model* and *time-varying rate model* in São Paulo, lead to larger number of deaths. In general, the impact of behavioural relaxation on deaths is much smaller than the impact of vaccination and NPIs in each region.

Table F. Death difference in a counterfactual without behavioural relaxation.

	Constant rate	Time-varying rate	Constant rate (vaccinated only)	Time-varying rate (vaccinated only)
British Columbia	-5 [-53,42]	-1 [-37,31]	4 [-34,39]	1 [-23,25]
Lombardy	-664 [-2384,-120]	-94 [-261,33]	-56 [-166,51]	-22 [-124,61]
London	-178 [-746,-35]	-19 [-77,28]	-1 [-48,49]	0 [-21,22]
São Paulo	-17399 [-31562,-5540]	-3508 [-15659,-158]	-702 [-1897,-144]	-318 [-1087,20]

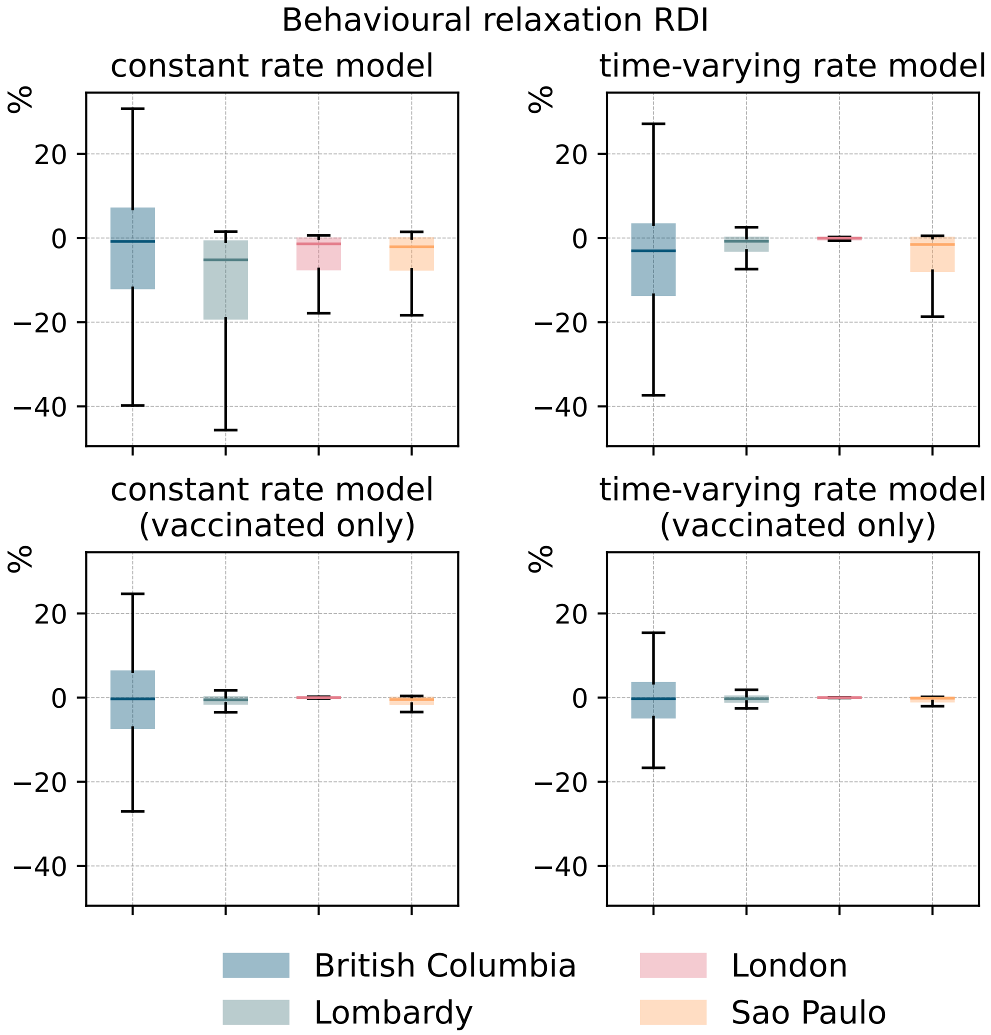

Fig L. Relative infection difference in counterfactual scenarios without behavioural relaxation. The fraction of total infections averted by behavioural relaxation. The box plots show the results of averted deaths in 1000 stochastic trajectories in each region. The horizontal line within each box marks the median value, while the top and bottom edges correspond to the 0.25 and 0.75 quartiles. The whiskers extend to the maximum and minimum values after removing the outliers that beyond the interquartile range.

5 Model calibration

5.1 AIC/BIC scores of models

In Table G we report the AIC scores of the five models. Smallest scores indicates a better performance of a model. As is shown, the baseline reports the smallest scores in the three regions (British Columbia, Lombardy, and London) out of four, while only one behavioural model (*time-varying rate model*) reports the smallest AIC score in São Paulo.

Similarly, we show the BIC scores of the five models in Table H. Smallest scores indicates a better performance of a model. As is shown, the baseline reports the smallest scores in all the regions, though in Lombardy *constant rate model* has very similar BIC scores to the baseline model.

Table G. AIC scores of models.

	Baseline	Constant rate	Time-varying rate	Constant rate (vaccinated only)	Time-varying rate (vaccinated only)
British Columbia	**202.6**	217.2	217.0	209.9	210.1
Lombardy	408.0	**403.4**	411.4	414.1	411.9
London	**273.5**	280.8	281.2	279.5	278.3
São Paulo	**498.3**	501.7	501.5	503.5	503.0

Table H. BIC scores of models.

	Baseline	Constant rate	Time-varying rate	Constant rate (vaccinated only)	Time-varying rate (vaccinated only)
British Columbia	**211.4**	230.4	230.2	223.1	223.3
Lombardy	**418.0**	418.4	426.4	429.0	426.9
London	**280.8**	292.5	292.9	291.2	290.0
São Paulo	**509.2**	517.3	517.1	519.1	518.5

5.2 Sensitivity analysis of AIC weight

We re-compute the AIC weights removing the last 1, 2, 3, 4 week(s) in the trajectories. The results are shown in Tables. I-L. The models' performance in terms of AIC weights is robust. *Constant rate model* is the most likely model in Lombardy and the baseline in the other three regions in all cases.

Table I. AIC weights of models of removing the last 1 week.

	Baseline	Constant rate	Time-varying rate	Constant rate (vaccinated only)	Time-varying rate (vaccinated only)
British Columbia	**0.95**	0.00	0.00	0.02	0.02
Lombardy	0.08	**0.88**	0.02	0.00	0.01
London	**0.84**	0.02	0.02	0.04	0.08
São Paulo	**0.64**	0.12	0.13	0.05	0.06

5.3 Sensitivity analysis of BIC

We computed also the BIC to measure models' performance. Similar to AIC, BIC also consider both fit and complexity of models, however, BIC prefers simpler models than AIC as it penalize the complexity more than AIC. The BIC weights of models are shown in Tables M-Q. Table M displays the BIC weights computed using complete trajectories, while Tables N, O, P, and Q show the results of sensitivity tests where we removed the last 1, 2, 3, 4 week(s) from the trajectories respectively. *Constant rate model* in

Table J. AIC weights of models of removing the last 2 weeks.

	Baseline	Constant rate	Time-varying rate	Constant rate (vaccinated only)	Time-varying rate (vaccinated only)
British Columbia	**0.95**	0.00	0.00	0.02	0.02
Lombardy	0.08	**0.89**	0.02	0.00	0.01
London	**0.84**	0.02	0.02	0.04	0.08
São Paulo	**0.64**	0.12	0.13	0.05	0.06

Table K. AIC weights of models of removing the last 3 weeks.

	Baseline	Constant rate	Time-varying rate	Constant rate (vaccinated only)	Time-varying rate (vaccinated only)
British Columbia	**0.95**	0.00	0.00	0.02	0.02
Lombardy	0.07	**0.89**	0.02	0.00	0.01
London	**0.84**	0.02	0.02	0.04	0.07
São Paulo	**0.64**	0.12	0.13	0.05	0.06

Table L. AIC weights of models of removing the last 4 weeks.

	Baseline	Constant rate	Time-varying rate	Constant rate (vaccinated only)	Time-varying rate (vaccinated only)
British Columbia	**0.95**	0.00	0.00	0.02	0.02
Lombardy	0.08	**0.88**	0.02	0.00	0.01
London	**0.84**	0.02	0.02	0.04	0.07
São Paulo	**0.65**	0.11	0.13	0.05	0.06

Lombardy does not emerge as the most likely when considering BIC weights for either the complete trajectories or when excluding the last 1 data point. The difference between *constant rate model* and the baseline is however rather small. When we remove the last 2, 3, 4 points, *constant rate model* in Lombardy becomes the most likely model. In general, the results are robust across AIC and BIC weights and different temporal horizons of data.

Table M. BIC weights of models.

	Baseline	Constant rate	Time-varying rate	Constant rate (vaccinated only)	Time-varying rate (vaccinated only)
British Columbia	**0.99**	0.00	0.00	0.00	0.0
Lombardy	**0.54**	0.44	0.01	0.00	0.01
London	**0.98**	0.00	0.00	0.01	0.01
São Paulo	**0.95**	0.02	0.02	0.01	0.01

The results with removing the last 1 week:

Table N. BIC weights of models of removing the last 1 week.

	Baseline	Constant rate	Time-varying rate	Constant rate (vaccinated only)	Time-varying rate (vaccinated only)
British Columbia	**0.99**	0.00	0.00	0.00	0.00
Lombardy	**0.51**	0.47	0.01	0.00	0.01
London	**0.98**	0.00	0.00	0.01	0.01
São Paulo	**0.95**	0.02	0.02	0.01	0.01

The results with removing the last 2 weeks:

Table O. BIC weights of models of removing the last 2 weeks

	Baseline	Constant rate	Time-varying rate	Constant rate (vaccinated only)	Time-varying rate (vaccinated only)
British Columbia	**0.99**	0.00	0.00	0.00	0.00
Lombardy	0.48	**0.50**	0.01	0.00	0.01
London	**0.98**	0.00	0.00	0.01	0.01
São Paulo	**0.94**	0.02	0.02	0.01	0.01

The results with removing the last 3 weeks:

Table P. BIC weights of models of removing the last 3 weeks

	Baseline	Constant rate	Time-varying rate	Constant rate (vaccinated only)	Time-varying rate (vaccinated only)
British Columbia	**0.99**	0.00	0.00	0.00	0.00
Lombardy	0.46	**0.51**	0.01	0.00	0.01
London	**0.98**	0.00	0.00	0.01	0.01
São Paulo	**0.94**	0.02	0.02	0.01	0.01

The results with removing the last 4 weeks:

Table Q. BIC weights of models of removing the last 4 weeks

	Baseline	Constant rate	Time-varying rate	Constant rate (vaccinated only)	Time-varying rate (vaccinated only)
British Columbia	**0.99**	0.00	0.00	0.00	0.0
Lombardy	0.48	**0.50**	0.01	0.00	0.01
London	**0.98**	0.00	0.00	0.01	0.01
São Paulo	**0.94**	0.02	0.02	0.01	0.01

5.4 Posterior distributions of parameters

In this section, we present the posterior distributions of the free parameters in our models computed via the Approximate Bayesian Computation-Sequential Monte Carlo (ABC-SMC). For each region, we plot the median values of the sampled parameters, along with the interquartile range (IQR) spanning from the first to the third quartile. Notably, the number of free parameters in the baseline model differs among regions due to the emergence of the second variant. Specifically, for the baseline model, in British Columbia and Lombardy, there are six free parameters to be calibrated: the reproductive number R_0, the delay time in reporting deaths Δ, the initial fraction of infected individuals i_{ini}, the initial fraction of recovered individuals r_{ini}, the adjustment of the start date of the simulation of epidemic Δt, and the adjustment of the introduction date of a VOC Δt_{var}. For London we have five free parameters as above, excluding Δt_{var}, as London experienced only one strain during the simulation period. In São Paulo, seven parameters are calibrated, the above six, as for British Columbia and Lombardy, and additionally the relative transmissibility (σ) of the Delta variant compared to the Gamma variant. As mentioned in the main text, this relative transmissibility for Alpha is fixed at 1.5 for British Columbia and Lombardy. Across all regions, the behavioural models consistently incorporate three additional behavioural parameters: the behavioural transition parameters α and γ, as well as the relative infection probability r of non-compliant individuals.

The posterior distributions of the four regions are shown in Figs. M-P. We compare the posteriors across the regions. For the reproductive number R_0, the calibrated values span from 1.0 to 2.5. London exhibits the highest R_0 across all models, due to the circulation is Alpha variant at the beginning time of

our simulations. British Columbia and Lombardy instead reports lower ranges of R_0, corresponding to the circulation of the wild type in these two regions. Furthermore, these estimated posteriors of R_0 are in line with previous studies. The posterior estimate for R_0 in British Columbia is 1.3 (90% CI: $[1.1 - 1.5]$), which is in line with Ref. [15] that reports values above 1 and below 1.5 in British Columbia in October 2020. The posterior estimate for R_0 in Lombardy is 1.26 (90% CI: $[1.04 - 1.80]$), consistent with the Ref. [16] suggesting values around 1.25 at the end of October 2020. The estimation is 1.8 (90% CI: $[1.4 - 2.3]$) in São Paulo which is in line with Ref. [17] reporting values in Brazil in January between 1.5 and 2. Nevertheless, we would like to note how the calibrated values of R_0 are affected by the model structure and specifically by how the force of infection is described. Hence, comparisons of particular values across different models should be interpreted carefully [18]. The delay in reporting deaths (Δ) ranges from 23 to 64 days across the four regions. British Columbia shows a longer delay. In contrast, Lombardy, shows a shorter delay. This difference may be due to difference in healthcare reporting systems. Regarding the posteriors of initial fraction of infected individuals i_{ini}, São Paulo shows the largest fraction with a median of 0.0048 (baseline model) across the four regions. The posteriors of the fraction of recovered r_{ini} shows the highest value with a median of 0.294 (baseline model) in Lombardy. This is consistent with the fact that Lombardy experienced the highest mortality rate across the four regions till the start date in our simulation. The medians of the adjustment for the start date of simulations (Δt) are 2, 6, 3, 4 weeks in British Columbia, Lombardy, London, and São Paulo. These figures suggest the best fit epidemic starting dates are 2, 6, 3, 4 weeks prior to the baseline date $t*$ set in section Materials and Methods in the main text. The adjustment of the introduction date of a VOC Δ_{var} shows a median of 11 in days in British Columbia and Lombardy, while it shows a median of 38 days in São Paulo. Analogously, these figures suggest the best fit dates at which we introduce a second variant with a fraction of 0.01 of infections in British Columbia, Lombardy, and São Paulo are 11, 11, 38 days prior to the baseline date t^*_{var} set in section Materials and Methods in the main text.

Regarding the parameters in the behavioural models, parameters that control behavioural rate α and γ are sampled in a logarithm scale. We transform the sampled values by $exp(x)$. The maximum values of medians of both α and γ are around 0.1 across the four regions. According to Fig. J, given α/γ equal to 0.1, the behavioural trasition rates from NC to C or from C to NC are under 0.2, exibiting a small behavioural values. This leads to the RDD/RDI results that behavioural mechanisms do not have a large impact on deaths and infections. Besides, São Paulo reports the largest α and the lowest γ compared to the other three regions, which is align with the largest RDD in São Paulo. The relative infection probability r of non-compliant individuals are similar across the regions within the range between 1.1 and 1.4, suggesting that non-compliant individuals have 1.1 to 1.4 times higher infection probablity than compliant individuals estimated by our models.

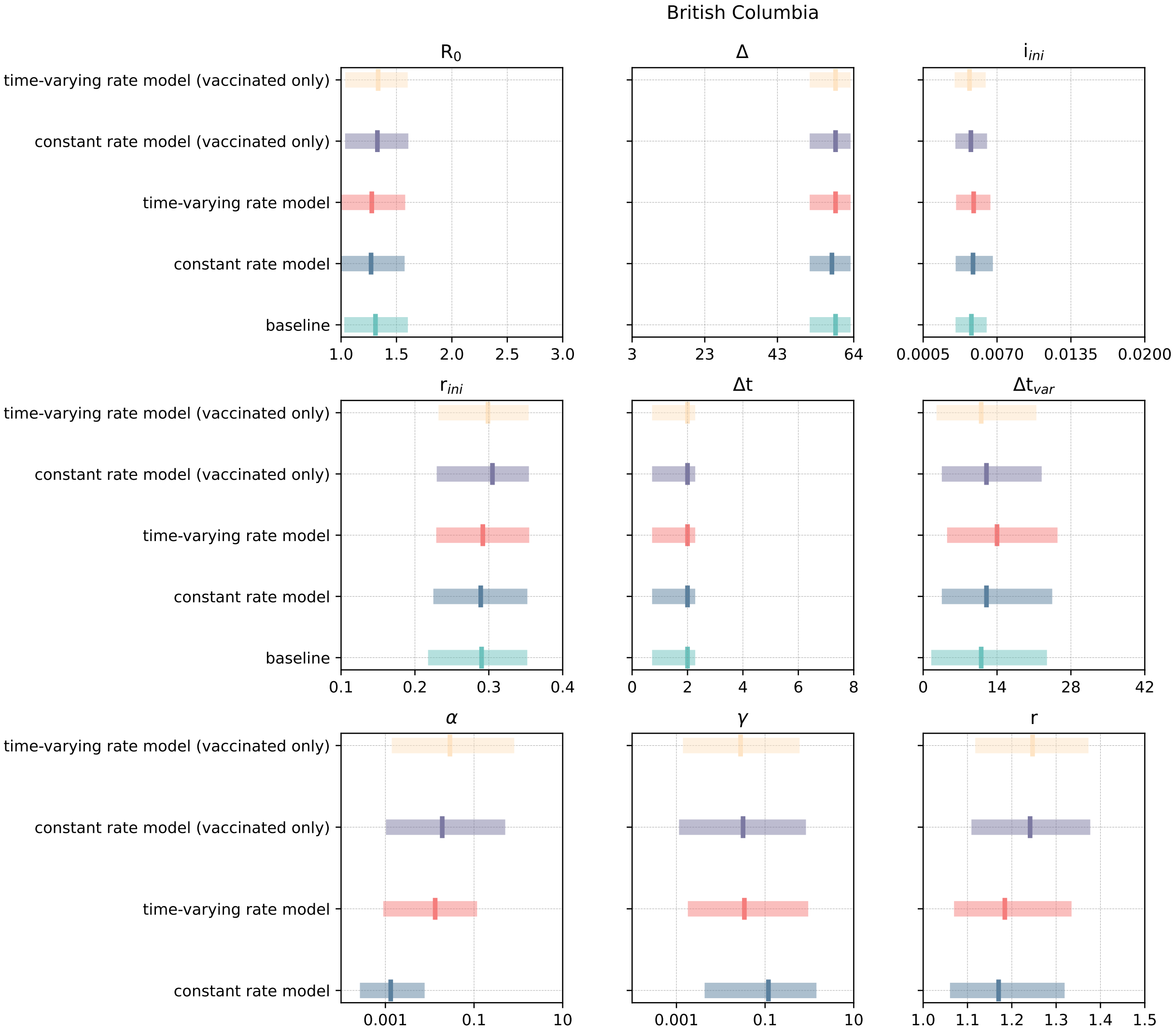

Fig M. Posterior distributions of calibrated parameters for British Columbia. We plot the median with the range between first quartile and third quartile. The first sixth parameters, R_0, Δ, i_{ini}, r_{ini}, Δt, Δt_{var} are calibrated in all the five models. α, β, and r are behavioural parameters only in behavioural models.

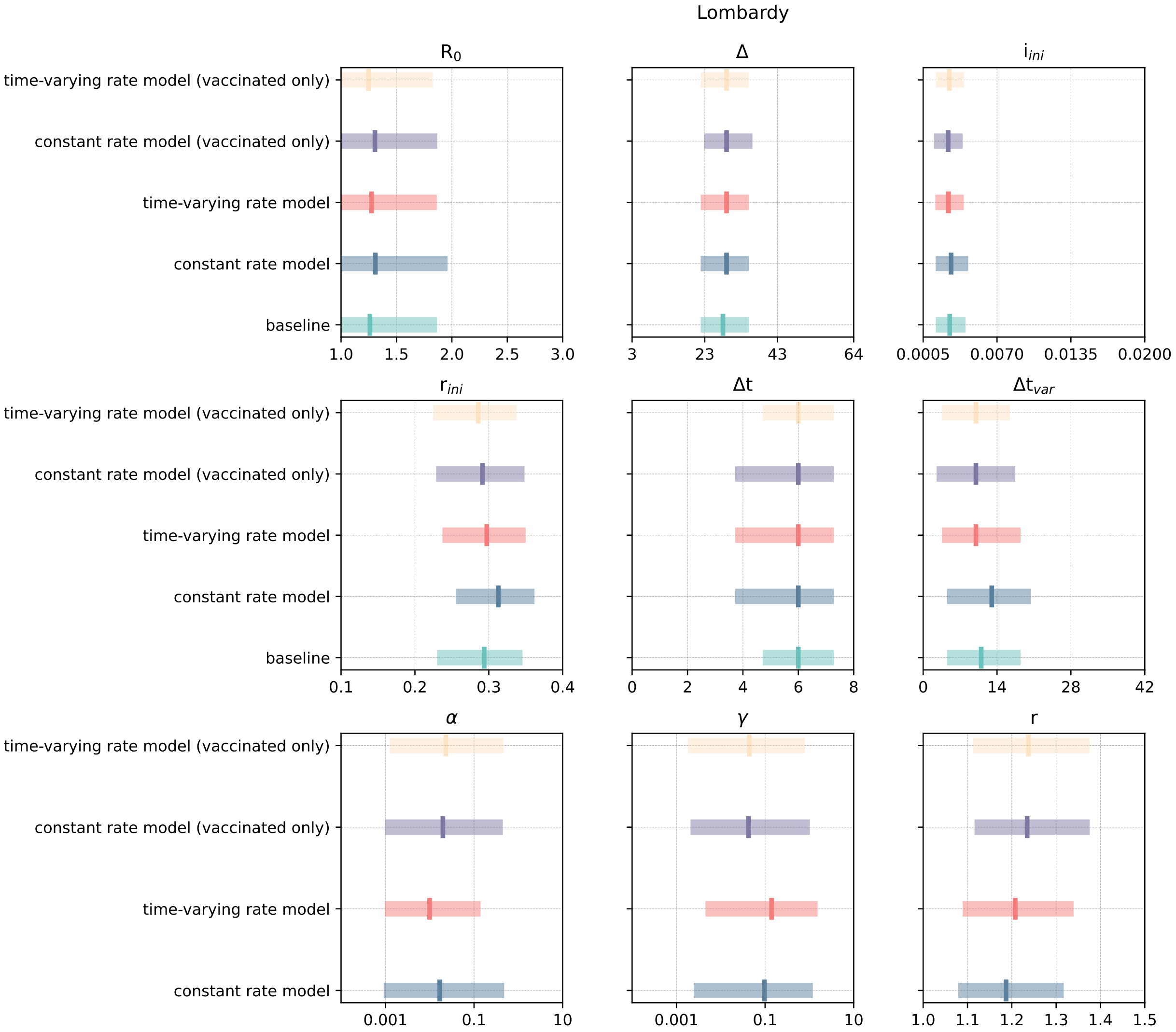

Fig N. Posterior distributions of calibrated parameters for Lombardy. We plot the median with the range between the first quartile and the third quartile. The first sixth parameters, R_0, Δ, i_{ini}, r_{ini}, Δt, Δt_{var} are calibrated in all the five models. α, β, and r are behavioural parameters only in behavioural models.

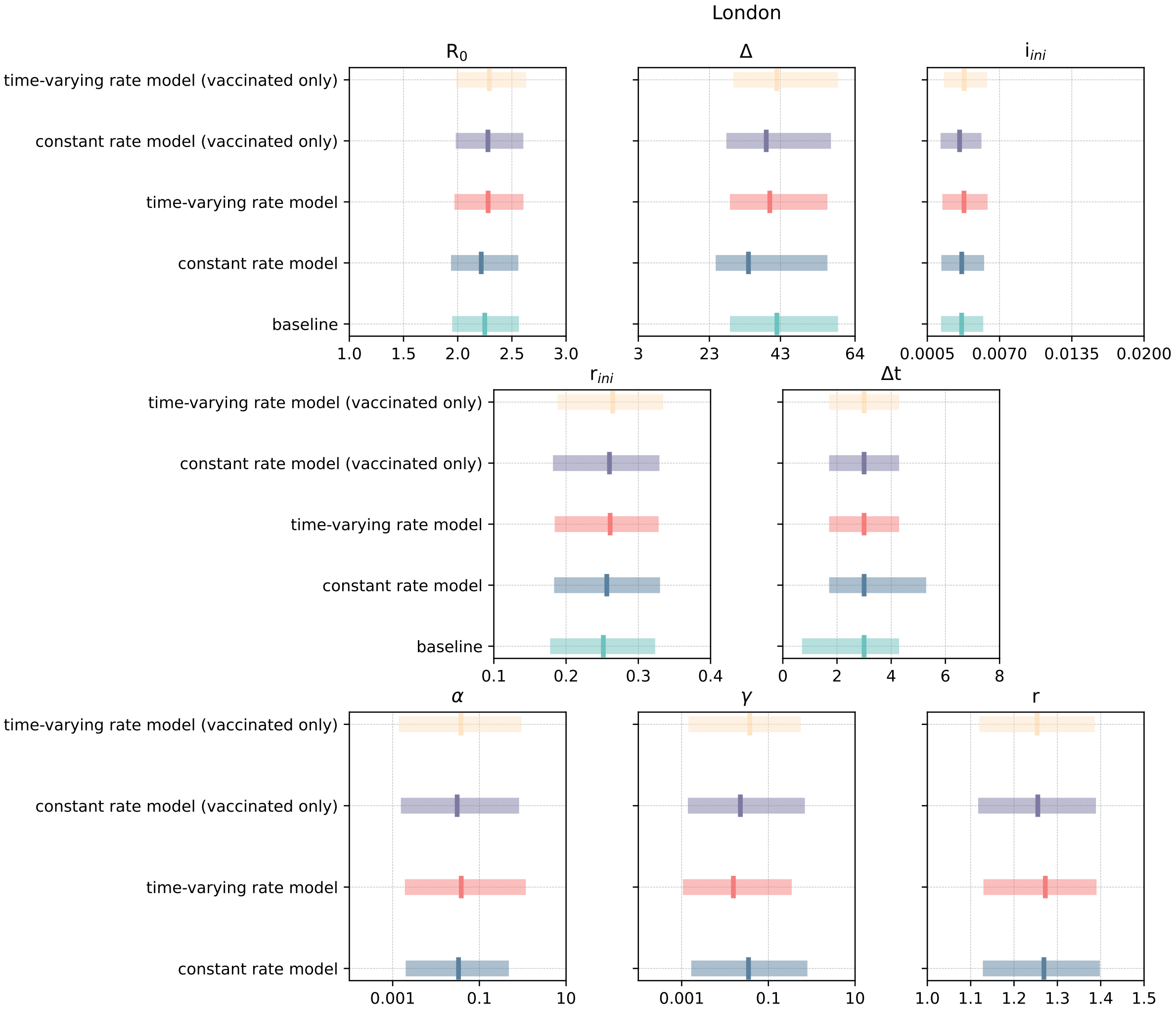

Fig O. Posterior distributions of calibrated parameters for London. We plot the median with the range between the first quartile and the third quartile. The first sixth parameters, R_0, Δ, i_{ini}, r_{ini}, Δt are calibrated in all the five models. α, β, and r are behavioural parameters only in behavioural models.

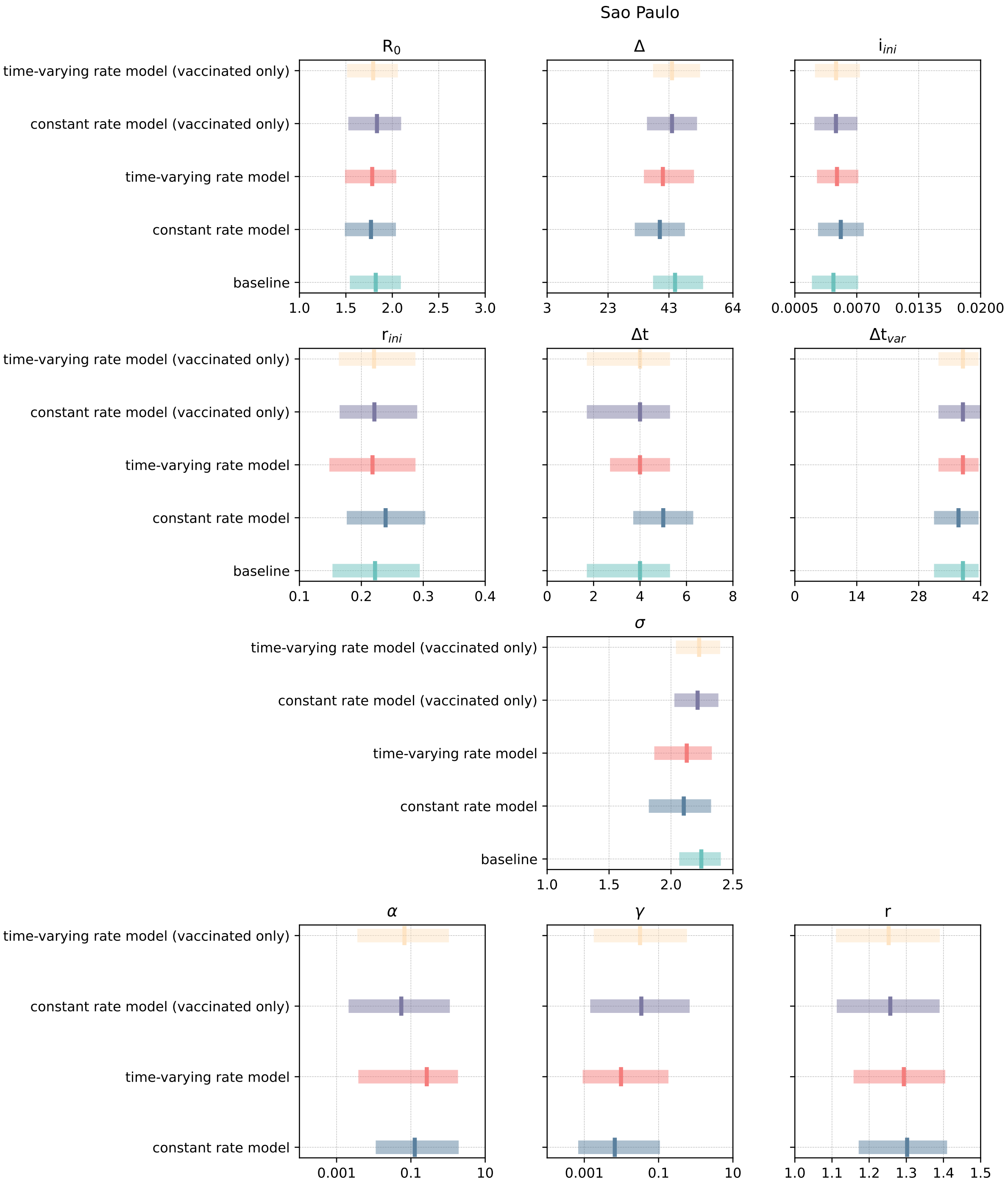

Fig P. Posterior distributions of calibrated parameters for São Paulo. We plot the median with the range between the first quartile and third quartile. The first sixth parameters, R_0, Δ, i_{ini}, r_{ini}, Δt, Δt_{var}, σ are calibrated in all the five models. α, β, and r are behavioural parameters only in behavioural models.

6 Bibliography

References

[1] Robert Verity, Lucy C Okell, Ilaria Dorigatti, Peter Winskill, Charles Whittaker, Natsuko Imai, Gina Cuomo-Dannenburg, Hayley Thompson, Patrick GT Walker, Han Fu, et al. Estimates of the severity of coronavirus disease 2019: a model-based analysis. *The Lancet infectious diseases*, 20(6):669–677, 2020.

[2] Kiesha Prem, Alex R Cook, and Mark Jit. Projecting social contact matrices in 152 countries using contact surveys and demographic data. *PLoS computational biology*, 13(9):e1005697, 2017.

[3] Pauline Van den Driessche. Reproduction numbers of infectious disease models. *Infectious disease modelling*, 2(3):288–303, 2017.

[4] João Viana, Christiaan H van Dorp, Ana Nunes, Manuel C Gomes, Michiel van Boven, Mirjam E Kretzschmar, Marc Veldhoen, and Ganna Rozhnova. Controlling the pandemic during the sars-cov-2 vaccination rollout. *Nature communications*, 12(1):3674, 2021.

[5] Jyotirmoy Roy, Samuel M Heath, Shiyan Wang, and Doraiswami Ramkrishna. Modeling covid-19 transmission between age groups in the united states considering virus mutations, vaccinations, and reinfection. *Scientific Reports*, 12(1):20098, 2022.

[6] Nicolò Gozzi, Matteo Chinazzi, Natalie E Dean, Ira M Longini Jr, M Elizabeth Halloran, Nicola Perra, and Alessandro Vespignani. Estimating the impact of covid-19 vaccine inequities: a modeling study. *Nature Communications*, 14(1):3272, 2023.

[7] Khaiwal Ravindra, Vivek Singh Malik, Bijaya K Padhi, Sonu Goel, and Madhu Gupta. Asymptomatic infection and transmission of covid-19 among clusters: systematic review and meta-analysis. *Public Health*, 203:100–109, 2022.

[8] Dhiraj Kumar Hazra, Bhalchandra S Pujari, Snehal M Shekatkar, Farhina Mozaffer, Sitabhra Sinha, Vishwesha Guttal, Pinaki Chaudhuri, and Gautam I Menon. Modelling the first wave of covid-19 in india. *PLoS computational biology*, 18(10):e1010632, 2022.

[9] Hongjun Zhao, Xiaoxiao Lu, Yibin Deng, Yujin Tang, and Jiachun Lu. Covid-19: asymptomatic carrier transmission is an underestimated problem. *Epidemiology & Infection*, 148:e116, 2020.

[10] Statistics canada. table 17-10-0005-01 population estimates on july 1, by age and gender. `DOI:https://doi.org/10.25318/1710000501-eng`.

[11] Italian national institute of statistics. resident population on 1st january: By age. `DOI:http://dati.istat.it/Index.aspx?QueryId=42869\&lang=en\#`.

[12] Nomis, office for national statistics (ons). population estimates - local authority based by five year age band. `DOI:https://www.nomisweb.co.uk/datasets/pestnew`.

[13] Brazilian institute of geography and statistics. tables - 2018 population projections for brazil and federation units by sex and age: 2010-2060. `DOI:https://www.ibge.gov.br/en/statistics/social/population/18176-population-projection.html?lang=en-GB`.

[14] Filippo Trentini, Adriana Manna, Nicoletta Balbo, Valentina Marziano, Giorgio Guzzetta, Samantha O'Dell, Allisandra G Kummer, Maria Litvinova, Stefano Merler, Marco Ajelli, et al. Investigating the relationship between interventions, contact patterns, and sars-cov-2 transmissibility. *Epidemics*, 40:100601, 2022.

[15] Isaac Chun-Hai Fung, Yuen Wai Hung, Sylvia K Ofori, Kamalich Muniz-Rodriguez, Po-Ying Lai, and Gerardo Chowell. Sars-cov-2 transmission in alberta, british columbia, and ontario, canada, december 25, 2019, to december 1, 2020. *Disaster medicine and public health preparedness*, 16(6):2428–2437, 2022.

[16] Mattia Manica, Giorgio Guzzetta, Flavia Riccardo, Antonio Valenti, Piero Poletti, Valentina Marziano, Filippo Trentini, Xanthi Andrianou, Alberto Mateo-Urdiales, Martina Del Manso, et al. Impact of tiered restrictions on human activities and the epidemiology of the second wave of covid-19 in italy. *Nature Communications*, 12(1):4570, 2021.

[17] Cecília Artico Banho, Beatriz de Carvalho Marques, Lívia Sacchetto, Ana Karoline Sepedro Lima, Maisa Carla Pereira Parra, Alex Ranieri Jeronimo Lima, Gabriela Ribeiro, Antonio Jorge Martins, Claudia Renata dos Santos Barros, Maria Carolina Elias, et al. Dynamic clade transitions and the influence of vaccination on the spatiotemporal circulation of sars-cov-2 variants. *npj Vaccines*, 9(1):145, 2024.

[18] Nicolò Gozzi, Nicola Perra, and Alessandro Vespignani. Comparative evaluation of behavioral-epidemic models using covid-19 data. *medRxiv*, pages 2024–11, 2024.
