# Supplementary material for: Estimating behavioural relaxation induced by COVID-19 vaccines in the first months of their rollout
Source: PLoS Comput Biol. 2025 Jul 7;21(7):e1013266. doi: 10.1371/journal.pcbi.1013266 (PMC12258572; doi:10.1371/journal.pcbi.1013266)
Supplement: Attachment — Submitted filename: review_Li_Gozzi_Perra_2025.pdf [file pcbi.1013266.s002.pdf]

Summary of the research and overall impression:

This manuscript investigates the hypothesis that the availability of COVID-19 vaccines during the first rollout influenced the adoption of non-pharmaceutical interventions (NPIs) due to behavioral relaxation. Behavioral relaxation is modelled by incorporating additional compartments that capture variations in adherence to NPIs into a baseline model. The study concludes that the addition of behavioral compartments does not necessarily lead to a strong improvement in model accuracy, highlights the issue of non-identifiability.

Although the study advances the field by fitting the model to real-world data, the transition mechanisms between compliant and non-compliant compartments rely on assumptions that could not yet be empirically validated. To address this limitation, the authors explore four possible mechanisms. However, I have some concerns regarding the structure of the models, which I outline below. Finally, both the writing and the presentation of results could be improved, as some key aspects remain unclear in the main text, limiting accessibility.

Major comments

Results

Line 112. *Google mobility data were used to modulate the synthetic contact matrices as extracted by Prem et al. 2017 over time.* Some more details should be provided on the construction of the matrix, including the population data used for the four regions and the mobility reduction applied over time and across different settings. Did you compare these matrices with empirical ones if available? [Trentini et al., *Epidemics* (2022) for Lombardy].

Line 127. I would appreciate a clearer and more compelling justification for the assumptions behind the transition function from compliant to non-compliant behaviors. To enhance readability, it might be helpful to associate each model with a key words that encodes its main feature. Synthesizing the main differences across the models in a table would help readers quickly catch key distinctions.

Line 128. Variations in adherence to NPIs translate in a higher force of infection for not-compliant individuals, which is modulated by the factor r. Some evidence suggests an age-gradient in both risk perception and compliance with NPIs. I wonder if the parameter r should also be age-dependent to better reflect these differences.

Line 142. *Only susceptible vaccinated individuals might transition to non-compliant compartments.* I expect that it would be more dangerous in terms of deaths if infected people would not compliant. Could you please provide more compelling justification for that?

Line 289. *The peak height of weekly deaths in British Columbia is more than five time smaller than the other regions. Hence, the NPIs in place, and their adoption, successfully managed to reduce the burden of the disease more than in the other locations.* Something else? Connectivity, population density?

Line 306. *Furthermore, the comparison between the two highlights the potential negative effects of behavioural relaxation mechanisms.* Could you please clarify how this sentence is supported? I have some difficulty in the understanding.

Discussion

Line 415. *Indeed, behavioural relaxation might have been more prevalent but not to the levels needed to affect mortality levels observed at a macroscopic scale.* I wonder whether fitting the model to additional data - such as behavioral data on compliance besides epidemiological data - could lead to more robust conclusions.

Methods

Line 456. It is unclear to me whether the 16 different age classes were introduced specifically to simulate the staggered vaccination rollout over time. In Figure 1, only a single vaccination opening date is reported. Could the model be simplified by distinguishing only between the elderly and the adult population for vaccination schedule? If so, the reduced number of age classes would allow for a more precise description of disease progression without increasing model complexity.

Line 464. In the proposed model, no distinction is made between asymptomatic and symptomatic individuals. I assume this choice was made to simplify the vaccination modeling, especially given the large number of age classes. However, I would expect that individuals with severe symptoms have a higher probability of death compared to asymptomatic cases. Consequently, the infection fatality ratio (IFR) for asymptomatic cases should be lower than for symptomatic ones. Is the IFR estimate used in the model valid both for symptomatic and asymptomatic individuals? In the reference cited, IFR seems to be derived from a cohort of hospitalized individuals.

Line 464. If I understand correctly, isolation of positive individuals is not explicitly modelled. Could you clarify how this aspect is accounted for? Are you assuming that the effect is absorbed into the mobility reduction?

Line 482. Why didn't you consider the effectiveness against transmission?

Line 488. If I understand correctly, the contact matrix remains the same for both compliant and non-compliant individuals. However, a recent survey in England found that 48.3% of participants reported meeting with friends or family more frequently after vaccination [Antonopoulou, ResearchSquare 2024]. Should more specific behavioral changes be accounted for ?

Minor comments

Intro

Line 2:31. The references range from the Global North to a global perspective to very specific countries, which I feel somewhat confusing. Moreover, reference [6] refers to LMICs, which are not necessarily part of the Global North, and the same applies to Vietnam.

Line 54. Could you please clarify what is included under *protective behavior*? Does it include mask-wearing and social distancing, or are there other behaviors considered as well?

Results

Line 182. *We use this data to compute the contact levels during the Pandemic with respect to a pre-Pandemic baseline.* How contact levels comparison is derived? It doesn't seem to be described in the methods section.

Methods

Figure 5. The use of "rV" for vaccination rates and "r" for the multiplying factor of the force of infection could create confusion. I suggest using an alternative notation to distinguish the two.

Table 4. Are the estimated R0s comparable with available estimates?

Miscellaneous remarks (other points)

The results are sometimes presented in a different order compared to the panels. To facilitate reading, could you please adjust the order to align them more clearly?

The first part of the results on the impact of vaccinations and NPIs seems to me a bit out of scope if the primary focus is on assessing behavioral relaxation.

Line 64:88. In my opinion, the preview of the results is misplaced. According to the journal guidelines, the introduction should conclude with a brief statement outlining the overall aim of the study and a comment on whether that aim was achieved.

Line 533. Ref missing in the text.
