# Supplementary material for: Estimating behavioural relaxation induced by COVID-19 vaccines in the first months of their rollout
Source: PLoS Comput Biol. 2025 Jul 7;21(7):e1013266. doi: 10.1371/journal.pcbi.1013266 (PMC12258572; doi:10.1371/journal.pcbi.1013266)
Supplement: Attachment — Submitted filename: behav_vaccine PlosCB rebuttal.pdf [file pcbi.1013266.s003.pdf]

Dear Editor,

We are pleased to resubmit our revised manuscript entitled "*Estimating behavioural relaxation induced by COVID-19 vaccines in the first months of their rollout*", for consideration in PLoS Computational Biology.

We are truly grateful to the reviewers for the constructive feedback and the positive evaluation of our work. As detailed in our point-by-point response below, we have considered all comments and suggestions. Here, we summarize the key revisions and additional analysis that have been made to the manuscript:

1. We have re-run all models considering a more detailed compartmentalization, i.e., SLPIARD, which includes presymptomatic and asymptomatic individuals. Interestingly, the results obtained are consistent with those already reported. We have added these new findings in the Supplementary Information as additional sensitivity analysis.
2. We added additional explanations and links to the relevant literature to provide clearer support for models' assumptions.
3. We revised the writing of the whole manuscript addressing all comments raised by both reviewers to improve clarity and readability.

We believe that the additional runs and editing have strengthened the manuscript and addressed all reviewers' comments. We appreciate your consideration of our revised submission, and we dare to hope that the manuscript is now suitable for publication in PLoS Computational Biology.

Thank you in advance for your consideration.

Sincerely yours,

Yuhan Li, Nicolò Gozzi, Nicola Perra

Point-by-point response

Below we provide a rebuttal to all points raised by the referees. We report their comments, verbatim, in red and our replies in blue.

Reviewer #1

Summary of the research and overall impression:

This manuscript investigates the hypothesis that the availability of COVID-19 vaccines during the first rollout influenced the adoption of non-pharmaceutical interventions (NPIs) due to behavioral relaxation. Behavioral relaxation is modelled by incorporating additional compartments that capture variations in adherence to NPIs into a baseline model. The study concludes that the addition of behavioral compartments does not necessarily lead to a strong improvement in model accuracy, highlights the issue of non-identifiability.

Although the study advances the field by fitting the model to real-world data, the transition mechanisms between compliant and non-compliant compartments rely on assumptions that could not yet be empirically validated. To address this limitation, the authors explore four possible mechanisms. However, I have some concerns regarding the structure of the models, which I outline below. Finally, both the writing and the presentation of results could be improved, as some key aspects remain unclear in the main text, limiting accessibility.

We thank the reviewer for all insightful comments and suggestions which have helped us improve the manuscript. We addressed all the comments and suggestions below.

Major comments Results

Line 112. *Google mobility data were used to modulate the synthetic contact matrices as extracted by Prem et al. 2017 over time.* Some more details should be provided on the construction of the matrix, including the population data used for the four regions and the mobility reduction applied over time and across different settings. Did you compare these matrices with empirical ones if available? [Trentini et al., *Epidemics* (2022) for Lombardy].

We thank the reviewer for the comments and questions.

We took the contact matrices from Prem et al. 2017. Hence, we did not construct them ourselves. We assume these matrices to be a good presentation of contact structures

before the COVID-19 Pandemic. We modify them, as a function of time, to account for the effects of NPIs during the Pandemic by using the Google Mobility Reports and the Oxford Coronavirus Government Response Tracker. To clarify these important points, in the revised manuscript we provided more details (see lines 533-559 in Methods section). Moreover, to provide more information about the changes of contacts over time, we added a new subsection entitled 'Spectral radius of contact matrices as function of time' in the Supplementary Information.

We thank the reviewer for pointing out the relevant literature. We compared Lombardy's weekly contacts with those reported by Trentini et al. We note however that the paper does not provide the matrices. Hence, our comparison is limited to the general description reported by the authors.
The maximum variation in the total number of contacts we estimate with our approach is 37%. This change is computed with respect to the same baseline period defined by Trentini et al. Interestingly, our estimates are in line with their results. Indeed, they report a 16%, 30% and 42% reduction in daily contacts under mild, moderate, and strong restrictions (which, similarly to the tier system in the UK, were alternated during the fall of 2020 based on the epidemic burden).
Trentini et al compared their contact matrix variations with Google mobility data. They performed a correlation analysis finding positive correlations of 0.3 (considering contacts in workplaces), 0.87 (in retail and recreation), and 0.8 (in transit stations). In the revised manuscript we have added a reference to the aforementioned work, and added a section in the Supplementary Information where we discuss the qualitative comparison between the contact matrices. Please see lines 555-557 in the main text and section 2.4 in Supplementary Information for more details.

Line 127. I would appreciate a clearer and more compelling justification for the assumptions behind the transition function from compliant to non-compliant behaviors. To enhance readability, it might be helpful to associate each model with a key words that encodes its main feature. Synthesizing the main differences across the models in a table would help readers quickly catch key distinctions.

We thank the reviewer for the comments and suggestions. The functions used to model the transition from compliant to non-compliant are taken from the literature. In particular, these transitions match those of the models that we aimed to compare and test against real data. The literature (i.e., Teslya et al. *Commun Med* 2022, Gozzi et al. *Plos Computational Biology* 2021) investigated two transition types. The first is the simplest one could imagine: a constant rate. The idea behind this transition function is that the start of the vaccination campaign might bring a part of the population to change behavior. The second assumes that the tendency towards non-compliance is a function of the fraction vaccinated population. The justification for the two transitions comes from the health

belief model which posits that behaviors are shaped by many factors including the perceived risks and susceptibility. In this context, both transition types assume that the start of vaccinations changed the risk perception and led to a relaxation of the adoption of NPIs. In the revised version of the manuscript, we have added a more clear justification about the transition functions used and several additional references as support (see lines 133-141).

Following the suggestion of the reviewer, we re-named the four behavioural models (models 1-4) respectively as ‘constant rate model’, ‘time-varying rate model’, ‘constant rate model (vaccinated only)’, ‘time-varying rate model (vaccinated only)’ (see lines 109-132). Specifically, the ‘constant rate model’ and the ‘time-varying rate model’ assume all individuals may become non-compliant, while the ‘(vaccinated only)’ versions assume that only vaccinated may become non-compliant. We summarized the features and differences of the models in Table 1 and revised the relevant parts in the Methods section (see lines 603-615).

Line 128. Variations in adherence to NPIs translate in a higher force of infection for not-compliant individuals, which is modulated by the factor r. Some evidence suggests an age-gradient in both risk perception and compliance with NPIs. I wonder if the parameter r should also be age-dependent to better reflect these differences.

We thank the reviewer for the comment. We acknowledge the heterogeneity in risk perception and levels of compliance across age groups. However, incorporating age-specific transition rates to (from) non-compliance, α_k (γ_k), and risk factors, r_k, would necessitate calibrating the three parameters for 16 age groups. This would significantly increase models' complexity and challenge the calibration procedure. In the revised version of the manuscript we have added a note in the limitations about this point (see lines 462-466).

Line 142. *Only susceptible vaccinated individuals might transition to non-compliant compartments.* I expect that it would be more dangerous in terms of deaths if infected people would not compliant. Could you please provide more compelling justification for that?

The reviewer is right. Indeed, one could imagine adding a compartment of infected non-compliant and having two different r factors: one for susceptible non-compliant, say r_s, and another one for infectious non-compliant, say r_i. In doing so, and neglecting for simplicity age-structure in what follows, the force of infection experienced by susceptibles would be proportional to $S(I + r_i I_{NC})$, where I_{NC} are infectious non-compliant. Furthermore, the same expression for susceptible non-compliant would be proportional to $S_{NC}(r_s I + r_s r_i I_{NC})$. This model is definitely of interest and something we might explore in

future work. However, we would like to point out how the time spent in the infectious compartment is rather short (2.5 days). Hence, the impact of allowing infectious individuals to be non-compliant is expected to be small with respect to the impact of non-compliance of susceptibles, at least in our settings. Additionally, the introduction of this new effect would come with an extra parameter which is likely to increase the issues of non-identifiability as reductions in r_i can be offset by increases in r_s and vice-versa. Nevertheless, in the revised version of the manuscript, we added a note about this in the limitations (see lines 466-470).

Line 289. *The peak height of weekly deaths in British Columbia is more than five time smaller than the other regions. Hence, the NPIs in place, and their adoption, successfully managed to reduce the burden of the disease more than in the other locations.* Something else? Connectivity, population density?

We thank the reviewer for raising these questions. In that part of the paper we estimated the effects of NPIs by computing the relative deaths difference (RDD) between simulations with and without NPIs. British Columbia emerged as the region with the largest RDD. In other words, relatively speaking, removing NPIs would have resulted in a worse outcome than in the other regions. We attributed this result to the much lower peak and burden of the disease in British Columbia. Indeed, given those conditions, removing NPIs would shift the system into a different dynamical regime. These results do not really explain the reasons why the peak in British Columbia was smaller to begin with. They only provide counterfactual estimates of the relative impact of NPIs. Hence, the second sentence we wrote "*Hence, the NPIs in place, and their adoption, successfully managed to reduce the burden of the disease more than in the other locations*" was misleading. We revised that part of the paper to clarify (see lines 300-302).

Out of curiosity and inspired by the reviewer's suggestion, we checked the population density of the four regions. Interestingly, the values, obtained from [1], are:

British Columbia: 6/km2
Lombardy: 421/km2
London: 5690/km2
Sao Paulo: 185/km2

So the reviewer's intuition was correct. British Columbia is by far the region with the smaller population density, which has most likely contributed to the smaller peak.

[1] Thomas Brinkhoff: City Population. DOI: https://www.citypopulation.de

Line 306. *Furthermore, the comparison between the two highlights the potential negative effects of behavioural relaxation mechanisms.* Could you please clarify how this sentence is supported? I have some difficulty in the understanding.

We thank the reviewer for raising this point. What we meant to say is that comparing the averted deaths in a scenario without vaccines and in a scenario without NPIs, shows how, at least in the early phases of the campaign, NPIs played a more important role than vaccines in controlling the spread. Hence, this suggests that behavioral relaxation (i.e., decreased adoption of NPIs) might cause a worsening of the epidemic burden. In the revised version of the manuscript we have changed the sentence adding a clearer explanation (see lines 306-311).

Discussion

Line 415. *Indeed, behavioural relaxation might have been more prevalent but not to the levels needed to affect mortality levels observed at a macroscopic scale.* I wonder whether fitting the model to additional data - such as behavioral data on compliance besides epidemiological data - could lead to more robust conclusions.

We thank the reviewer for raising this interesting point. This is indeed an intriguing direction that we aim to explore in a follow-up work, where behavioral data (such as aggregated mobility metrics) is used as an additional target variable in the calibration process. The intuition is that doing so might help to address some of the non-identifiability issues we have discussed (see lines 435-441), and improve the overall fit. Nonetheless, this is a non-trivial problem that requires significant methodological development and assumptions (e.g., which behavioral data to use, how do we map model output to such data). For this reason, while we absolutely agree on the importance of taking this step further, we believe that this effort deserves dedicated work. We have added a note about this important direction of future work in the discussion (see lines 441-446).

Methods

Line 456. It is unclear to me whether the 16 different age classes were introduced specifically to simulate the staggered vaccination rollout over time. In Figure 1, only a single vaccination opening date is reported. Could the model be simplified by distinguishing only between the elderly and the adult population for vaccination schedule? If so, the reduced number of age classes would allow for a more precise description of disease progression without increasing model complexity.

We thank the reviewer for the insightful comments and suggestions. We would like to first clarify why we introduce 16 age groups. The contact matrix utilized in our study is derived from Prem et al. (2017), which is structured into 16 distinct age groups. To ensure consistency in formulating the dynamic equations, we categorized the population into the

same 16 age groups. Regarding the vaccination rollout, we indeed apply different vaccination rates across age groups based on available data. To maintain consistency with the structure of the dynamic equations, we organized the vaccination data to match the 16 age groups defined in the contact matrix.

In response to the reviewer's concern about the single vaccination start date reported in Figure 1, we would like to clarify that our model simulates the vaccination process as follows: each day a fraction of susceptible (unvaccinated) individuals in age group *i* transitions to the vaccinated state based on the reported administered vaccine doses for that specific age group in the given region. For simplicity, in Figure 1, we show vaccine rates only for the total and 70+ population, despite using more granular vaccine data for different age groups in the simulations. To clarify the vaccination process in our model, we provided more details about how we simulate the vaccine rollout in the subsection 'Modelling vaccination' in Materials and Methods (see lines 519-524).

We acknowledge the reviewer's suggestion to simplify the model by distinguishing only between the elderly and adult population. However, since vaccine rates are derived directly from data, using age-specific vaccination data at a finer granularity does not increase the model complexity (in terms of free parameters). Furthermore, considering 1) the strong dependence between age and deaths for COVID-19, 2) that weekly deaths are our target variable, and 3) that finer age stratification allows simulating the impact of targeted NPIs on specific groups such as school closures, we believe that considering explicitly different age-groups helps increase the realism of the model and ultimately the fit.

Line 464. In the proposed model, no distinction is made between asymptomatic and symptomatic individuals. I assume this choice was made to simplify the vaccination modeling, especially given the large number of age classes. However, I would expect that individuals with severe symptoms have a higher probability of death compared to asymptomatic cases. Consequently, the infection fatality ratio (IFR) for asymptomatic cases should be lower than for symptomatic ones. Is the IFR estimate used in the model valid both for symptomatic and asymptomatic individuals? In the reference cited, IFR seems to be derived from a cohort of hospitalized individuals.

We thank the reviewer for the comments. Indeed, we adopted a simpler compartmentalization (i.e., SLIR) which does not include asymptomatic individuals. To address the reviewer's concern regarding the difference of symptomatic and asymptomatic individuals, we have conducted additional sensitivity analyses to the modeling framework by considering a SLPIAR compartmentalization which explicitly includes two new compartments: presymptomatic (P) and asymptomatic (A). In this new setup, we assume that latent individuals transit to a presymptomatic phase (P). Then, individuals become either asymptomatic (A) with a probability f or symptomatic with probability $1-f$. We

assume that asymptomatic individuals will not develop severe symptoms. They eventually recover, after the infectious period. Symptomatic individuals will either recover or die according to the age-stratified infection fatality rate. This modification allows us to capture the difference in IFR between asymptomatic and symptomatic individuals. We also assume that individuals in P and A are less infectious by a factor χ (χ<1).

We present the results of this SLPAIR compartmentalization in a new subsection of Supplementary Information (see the subsection 1.3 entitled "Sensitivity to the modeling framework"). We have also added a pointer in the discussion (see lines 478-482). Overall, we find the results consistent with those obtained considering a SLIR compartmentalization. Hence, the conclusions are robust to the choice of modeling framework.

Line 464. If I understand correctly, isolation of positive individuals is not explicitly modelled. Could you clarify how this aspect is accounted for? Are you assuming that the effect is absorbed into the mobility reduction?

We thank the reviewer for these questions. Yes, isolating positive individuals is not explicitly modelled. The models account for isolation only effectively by considering a short infectious period of 2.5 days. This is the period in which, on average, infected individuals can infect others. After, we assume that they either develop symptoms and isolate, or get tested and isolate. To clarify this aspect, we added a note in the material and method section when we describe the details of the models (see lines 508-511).

Line 482. Why didn't you consider the effectiveness against transmission?

We thank the reviewer for this insightful question. We did consider only one aspect of the effectiveness of vaccines against transmission (VE_T). Indeed, VE_T might be broken down in two distinct contributions: 1) the effectiveness against susceptibility (VE_S) and 2) against infectiousness (VE_i). The first considers the reduced risk of a vaccinated individual of becoming infected. The second accounts for the reduced infectivity of a vaccinated individual in case of a breakthrough infection. We considered only VE_S. We opted to neglect VE_i for two reasons. First, we did not find clear/accepted/consensus values for all the variants of concern considered. We did find estimates for the Alpha and Delta variants (Braeye et al. Vaccine 2023 and Lyngse et al. Nature Communications 2022) but not for Gamma. Second, in the regions under examination here, the large number of infections took place when the fraction of vaccinated individuals was still small. Hence, for simplicity we have chosen not to incorporate VE_i in our models. We have added a note about this in the limitations (see lines 452-457).

Line 488. If I understand correctly, the contact matrix remains the same for both compliant and non-compliant individuals. However, a recent survey in England found that 48.3% of participants reported meeting with friends or family more frequently after vaccination [Antonopoulou, ResearchSquare 2024]. Should more specific behavioral changes be accounted for ?

We thank the review for this comment. The contact matrix for both compliant and non-compliant individuals is indeed the same. We use contact matrices to characterize contact patterns across age groups. They do not account for differences in the contact patterns of compliant and non-compliant individuals. The increased social interactions, in line with the study mentioned by the reviewer, are modelled through our behavioral relaxation mechanisms, where non-compliant individuals have a higher infection risk multiplied by the factor r.

Furthermore, the study of Antonopoulou et al suggests age-specific non-compliant behaviors, which could be modeled using an age-specific factor, i.e., r_k. However, the study does not provide sufficient details to enable adjustments to the contact matrix at the level of age group interactions. It would be possible to construct more precise contact matrices for non-compliant individuals if we had data on non-compliant behaviors at the level of age group interactions. We hope to explore this in future work when the relevant data becomes available. We have added a reference to the paper in the introduction when we discuss empirical evidence of vaccine-induced behavioral relaxation (see lines 30-34, 132).

Minor comment

Intro

Line 2:31. The references range from the Global North to a global perspective to very specific countries, which I feel somewhat confusing. Moreover, reference [6] refers to LMICs, which are not necessarily part of the Global North, and the same applies to Vietnam.

We thank the reviewer for the comment. We aimed to provide a comprehensive background in different contexts by showing examples of specific countries. We apologize for the confusion caused by this. We revised the narrative of the Introduction.

Line 54. Could you please clarify what is included under *protective behavior*? Does it include mask-wearing and social distancing, or are there other behaviors considered as well?

We thank the reviewer for these questions. In our context, protective behaviours refer to a wide range of actions implemented by individuals such as mask-wearing, hygiene practices, and social distancing. We added a note to clarify the range of behaviors we have in mind in the introduction (see lines 21-24).

Results

Line 182. *We use this data to compute the contact levels during the Pandemic with respect to a pre-Pandemic baseline.* How contact levels comparison is derived? It doesn't seem to be described in the methods section.

We thank the reviewer for the comment. The contact levels compared to pre-Pandemic baseline are directly obtained from the Google Mobility Reports. According to Google, mobility on any given day is compared to a baseline. The baseline is defined as the median value from the 5-week period Jan 3-Feb 6, 2020. In the revised version of the manuscript, we added more details about the mobility data in the Methods section (see lines 541-544).

Methods

Figure 5. The use of "rV" for vaccination rates and "r" for the multiplying factor of the force of infection could create confusion. I suggest using an alternative notation to distinguish the two.

We thank the reviewer for the suggestion to improve the clarity. In the revised version of the manuscript we defined vaccination rates as ν. Please see the revised notation in Fig. 5.

Table 4. Are the estimated R0s comparable with available estimates?

We thank the reviewer for the question. Our results are consistent with prior work specifically focused on the second wave of the Pandemic. In particular:

- Fung, Isaac Chun-Hai et al. *Disaster medicine and public health preparedness* (2022), report values just above 1 and below 1.5 in British Columbia October 2020, which is in line with our posterior estimate of 1.3 (1.1-1.5, 90% CI) at the simulation starting point in the same month.
- Manica et al. *Nat Commun* (2021) suggests that the reproductive number in Lombardy was around 1.25 at the end of October 2020, in line with our posterior estimate of 1.26 (1.04-1.80, 90% CI) for that period.

- Banho et al. *npj Vaccines* (2022) report values for Brazil in January between 1.5 and 2. This is in line with our posterior estimate of 1.8 (1.4-2.3, 90% CI) at the simulation starting point in January.

Nevertheless, we would like to note how the calibrated values of R0 are affected by the model structure and specifically by how the force of infection is described. In a different, comparative work, we have shown how behavioral models might provide different estimates for R0 when fitted to the same epidemic data (Gozzi, N., Perra, N. and Vespignani, A., 2024. Comparative Evaluation of Behavioral-Epidemic Models Using COVID-19 Data. *medRxiv*, pp.2024-11). Hence, comparisons of particular values across different models should be interpreted carefully. We have added all the references and a discussion about the comparisons (see lines 216-227 in the main text and blue texts in section 5.4 in the Supplementary Information).

Miscellaneous remarks (other points)

The results are sometimes presented in a different order compared to the panels. To facilitate reading, could you please adjust the order to align them more clearly?

We thank the reviewer for the suggestion. In the revised manuscript, we have aligned the discussion and panels more carefully.

The first part of the results on the impact of vaccinations and NPIs seems to me a bit out of scope if the primary focus is on assessing behavioral relaxation.

We thank the reviewer for the comment. After consideration, we have opted to leave this part in the paper as it provides the reference needed to judge the potential impact of behavioral relaxation on diseases' burden. Moreover, both vaccination and NPIs are key ingredients of our narrative. As such, we believe that showing/quantifying their impact is needed.

Line 64:88. In my opinion, the preview of the results is misplaced. According to the journal guidelines, the introduction should conclude with a brief statement outlining the overall aim of the study and a comment on whether that aim was achieved.

We thank the review for the comment. We revised the Introduction as suggested. Please see lines 62-72 in the revised manuscript.

Line 533. Ref missing in the text.

We thank the reviewer for pointing out the missing reference. We reviewed the manuscript and corrected the issue.

Reviewer #2

I enjoyed reading this paper. Its research question has a high relevance to the field of public health - demonstration of impact of NPIs and vaccinations on reducing the burden of the pandemic and validation of assumptions about the effects of behaviors on epidemic dynamics. The tools used are relevant to the research question and were explained in sufficient detail. Separately, I would like to commend the lucidity of the narrative. I have only minor comments and recommend this work for publication.

We are glad of the positive assessment of the reviewer. Furthermore, we sincerely thank the referee for all comments and suggestions that helped us improve the presentation of the manuscript. We addressed all the comments and suggestions below.

1. One substantial but minor comment: in the methods, you mention that when NPIs are in place, while contacts outside of home are generally decreasing, you chose not to model the resulting increase in home contacts. I think it would be nice to include the discussion of how your conclusions about the effect of behaviour relaxation in the wake of vaccination rollout are affected by this assumption.

We thank the reviewer for raising this point. Indeed, we did not model changes in contacts at home. In other locations, we assume that changes in contacts scale quadratically with respect to the changes in mobility data. This is because Google Mobility Reports provide information about variations in “visits” to specific location types. Indeed, under an homogenous mixing assumption, the number of possible contacts in a given location is proportional to the square of the number of people visiting that location. It is unclear to which extent the link between the two quantities applies at home. We do expect some saturation effects which might also be region dependent. For this reason, in this and in many other previous papers, we do not model changes of contacts at home. In the revised version of the manuscript, we have added a note on the limitations about this point (see lines 470-475), and a more detailed discussion in the methods (see lines 552-557).

2. Further minor points, mostly cosmetic:

a. Frequently used 'firm minority'. I was not entirely sure about the meaning of this qualifier, propose to revise.

We thank the reviewer for the comment. By “firm minority” we meant to highlight how the calibrated models suggest that only a minority (i.e., <50%) of the population relaxed their protective behaviors. We removed ‘firm’ in the manuscript as it creates confusion.

3. frequently 'however' is not placed correctly, disrupting the sentence flaw, making comprehension more difficult. For instance, the first paragraph of Introduction: 'Especially in the first months of their rollout, however, vaccination efforts 4 have encountered many challenges due to logistical issues, limited stockpiles and 5 healthcare capacity, vaccine nationalism, and spotty acceptance [7].' i propose 'However, especially in the first months of their rollout, vaccination efforts..' This comment is applicable to several instances in the text.

We thank the reviewer for the suggestion. We have revised the manuscript to correct the use of the word ‘however’ throughout the manuscript.

4. The same paragraph - the introduction opens with description of the roll out in the Global North - can you explain why? Was it the first location which started the vaccination rollout? And why is the worldwide rollout restricted only to the Global North?

We thank the reviewer for the comment. We thought of opening with a mention of the “Global North” to highlight how rich countries started their rollout much earlier than others. However, as also mentioned by the first reviewer, our opening narrative was a bit confusing. In the revised version of the manuscript we have rephrased it.

5. Same paragraph: I was not clear on what is 'Vaccine nationalism'. Does this refer to vaccine stockpiling?

We thank the reviewer for the questions. Vaccine nationalism is a term often used to describe the unfortunate tendency of many countries to stockpile as many vaccines as possible without considering global needs. We have added a clarification (see lines 10-11).

6. Same paragraph, sentence 'The insufficient vaccination coverage in many areas proved inadequate to prevent subsequent waves, thus leading to increased disease burden and to the implementation of additional interventions to curb the spread of SARS-CoV-2.' Increased disease burden compared to what? Did you mean resurgence of new cases?

We thank the reviewer for this question. By "increased disease burden" we meant to say "increase in cases and deaths". We have revised the sentence to "The insufficient vaccination coverage in many areas proved inadequate to prevent subsequent waves and reduce both cases and deaths" (see lines 12-14).

Overall, the first paragraph has narrative flows: we keep moving back and forth between the reasons why vaccination rollout was not smooth, and the consequences of it.

We thank the reviewer for the comment. We intended to outline the various challenges of vaccination, which include limited resources, low acceptance and coverage, disparities across countries, and the emergence of new variants that can evade immune responses. We revised the text aiming to streamline the narrative (see lines 2-16).

Finally, the last sentence of the paragraph, I would argue that the prime goal of vaccines was to reduce morbidity and mortality, with the hope that the same would follow for the transmission.

We thank the reviewer for the comment. We agree that the primary goal of vaccines was to protect individuals from developing severe symptoms. However, we wish to highlight the considerable reduction in vaccine efficacy against new variants, which is also a critical factor considered in our models. We have revised the text to clarify (see lines 14-16).

7. Occasional incorrect usage of 'how'. Illustration: the second paragraph of Introduction: 'The results from these efforts suggest how behavioural relaxation could reduce the positive gains brought about by vaccines thus leading to higher disease burden.' Here 'that' would be more accurate. I overall counted at least 3 similar instances.

We thank the reviewer for the comment. We checked all sentences where we used 'how' and carefully considered whether the word was appropriate or not in the revised manuscript.

8. Third paragraph of the introduction, lines 46-48. What do you mean by simpler baseline? A model without behavioral component? I would say so directly.

We thank the reviewer for the suggestion. We revised 'simpler baseline' as 'simpler models that do not feature behavioural relaxation mechanisms' (see lines 51-52).

9. Line 53 'instead' is unnecessary. Frequently, 'instead' is not used correctly, hampering the flow of sentences.

We thank the reviewer for the comment. We checked all the sentences where we use 'instead' and revised them.

10. Paragraph starting on line 58 almost belongs entirely in the discussion.

We thank the reviewer for the comment. This point was raised also by reviewer #1. We have revised the introduction removing all parts that are more appropriate for the discussion. Please see lines 62-72 in the revised manuscript.

11. Line 72 - citation is needed.

We thank the reviewer for the suggestion. We added the reference for the statement at the original line 72, which has been moved from Introduction to Results (see lines 346-348).

12. Lines 132-133 Subsection Behavioural relaxation models, section Results. In this model, what drives individuals to become compliant or non-compliant? Is it other people holding these views? or is the term describing changes in compliant population given thus S^{c}' = \alpha S - gamma S_{c}?

We thank the reviewer for the comment. We have two different transition types. In constant rate models, the transition occurs at a constant rate. This is the simplest approach possible and stems from the idea that the start of the vaccination campaign might induce people to relax their behavior. In the time-varying rate models, the transition to non-compliant behaviour is a function of the fraction of the fraction vaccinated individuals and a free parameter. The reverse transition rate (from non-compliant to compliant) is a function of the number of reported daily deaths per 100K and a free parameter. In this case the tendency towards compliance is set by the fraction of the vaccinated population while the transition back is driven by a possible worsening of the Pandemic. Motivated also by a similar comment of the first reviewer, in the revised manuscript we added a more detailed description of the various models and renamed the models (see Table 1), providing clearer justifications and highlighting the assumptions (see lines 109-141).

13. Same paragraph, lines 141-142. In [24], only un-vaccinated people participated in compliance process. So in this sence, [24] is closer to models [1] and [2].

We thank the reviewer for the comment. We would like to clarify that in model 1 (now named as *constant rate model*) and model 2 (now named as *time-varying rate model*), both vaccinated and unvaccinated individuals might become non-compliant. In the model proposed in Ref. [24] instead, only vaccinated individuals might become non-compliant. Following Ref. [24], we developed model 3 (now named as *constant rate model (vaccinated only)*) and model 4 (now named as *time-varying rate model (vaccinated only)*). Hence, models 3 and 4 are closer to Ref. [24]. We provided clearer descriptions of behavioral models (see lines 109-132, 603-614).

Results

1. Lines 169-179 - what is meant by 'less intense'? rate of growth, magnitude, cumulative number?

We thank the reviewer for the comment. We meant to highlight that in British Columbia the peak of weekly deaths was more than five times lower compared to the other three regions. We have revised the sentence (see lines 169-170).

2. Lines 194-196 - Perhaps I am misunderstanding something, but would not the increase to levels beyond the pandemic levels result in value greater than 1 - but on the panel it is below 1?

We thank the reviewer for pointing out this. We apologize for the misleading statement. We meant to say that in Sao Paulo the mobility levels at the end of the observed time are much higher than that at the beginning of the observed time (not the pre-pandemic level). We aimed to emphasize the rebound trend toward pre-pandemic level. We have corrected the descriptions. Please see the revised sentence (see lines 193-196).

3. lines 364-365. I am not clear, if you remove relaxation mechanisms, what stays to make the models different from baseline? especially in the case where the rate of transition to and from compliance does not depend on epidemiological and immunological state of the population.

We thank the reviewer for the comment. The behavioural models are built upon the baseline framework, with the addition of behavioural relaxation mechanisms. Therefore yes, if these mechanisms are removed, the behavioural models reduce to the baseline.

However, the inclusion of behavioral relaxation mechanisms leads to a number of non-compliant individuals who are assumed to have a higher risk of infection, thereby influencing the models' outcomes, even in the case of constant transition rates.

Notaly, these transition rates are calibrated by fitting the models' outputs (i.e., weekly deaths) to surveillance data. This means that even constant rate mechanisms are informed by real epidemic data. We hope these clarify the reasoning behind the models and appreciate your feedback.

Methods

1. line 459 'follow' should be 'follows'
2. line 462 'as' should be 'to be'
3. line 464 'after' should 'subsequently'

We thank the reviewer for these suggestions. We have modified the manuscript accordingly.

4. Figure 5, in flow diagram what does subscript 'k' in 'lambda' refers to? Coincidentally 'lambda' does not seem to be defined, and we do not know the relative difference in ability to transmit of vaccinated vs unvaccinated individuals.

We thank the reviewer for the comments. We apologize for not introducing the force of infection λ_k before presenting it in the figure. In the revised manuscript, we added the definition for λ_k in the paragraph before showing the figure (see lines 500-502).

Regarding the transmission of vaccinated vs unvaccinated, we did consider only one aspect of the effectiveness of vaccines against transmission (VE_T). Indeed, VE_T might be broken down in two distinct contributions: 1) the effectiveness against susceptibility (VE_S) and 2) against infectiousness (VE_i). The first considers the reduced risk of a vaccinated individual of becoming infected. The second accounts for the reduced infectivity of a vaccinated individual in case of a breakthrough infection. We considered only VE_S. We opted to neglect VE_i for two reasons. First, we did not find clear/accepted/consensus values for all the variants of concern considered. We did find estimates for the Alpha and Delta variants (Braeye et al. Vaccine 2023 and Lyngse et al. Nature Communications 2022) but not for Gamma. Second, in the regions under examination here the larger number of infections took place when the fraction of vaccinated individuals was still small. Hence, for simplicity we have chosen not to incorporate VE_i in our models. We have added a note about this in the limitations (see lines 452-457).

5. Line 522: when you say 'allocating there 1%', do you mean they are moved from existing infections or injected extra? what is % refers to then?

We thank the reviewer for the comment. We meant to say that new individuals are added in that compartment. The % refers to the existing infected population. In the revised version of the manuscript we have clarified this point (see lines 571-573).

6. Table 4: overall vaccine efficacy for Delta variant is missing.

We thank the reviewer for the comment. The vaccine efficacy for the Delta variant was presented in table 6 after the Alpha variant. We revised the table highlighting the vaccine efficacy for the Delta variant in a separate line.
